# Molecular Insights into Mitochondrial Protein Translocation and Human Disease

**DOI:** 10.3390/genes12071031

**Published:** 2021-07-01

**Authors:** Eduardo Ruiz-Pesini, Julio Montoya, David Pacheu-Grau

**Affiliations:** 1 Departamento de Bioquímica, Biología Molecular y Celular, Universidad de Zaragoza. C/ Miguel Servet, 177, 50013 Zaragoza, Spain; eduruiz@unizar.es (E.R.-P.); jmontoya@unizar.es (J.M.); 2 Instituto de Investigación Sanitaria (IIS) de Aragón, Av. San Juan Bosco, 13, 50009 Zaragoza, Spain; 3 Centro de Investigaciones Biomédicas en Red de Enfermedades Raras (CIBERER), Av. Monforte de Lemos, 3-5, 28029 Madrid, Spain

**Keywords:** mitochondria, protein import, disease

## Abstract

In human mitochondria, mtDNA encodes for only 13 proteins, all components of the OXPHOS system. The rest of the mitochondrial components, which make up approximately 99% of its proteome, are encoded in the nuclear genome, synthesized in cytosolic ribosomes and imported into mitochondria. Different import machineries translocate mitochondrial precursors, depending on their nature and the final destination inside the organelle. The proper and coordinated function of these molecular pathways is critical for mitochondrial homeostasis. Here, we will review molecular details about these pathways, which components have been linked to human disease and future perspectives on the field to expand the genetic landscape of mitochondrial diseases.

## 1. Introduction

Mitochondrial DNA (mtDNA) is a small circular genome present in a variable copy number in almost all human cells and encodes for thirteen polypeptides, 22 tRNAs and 2 rRNAs. These thirteen proteins constitute the core components of the oxidative phosphorylation system (OXPHOS), located in the inner mitochondrial membrane and responsible for the production of the bulk of cellular energy in the form of ATP. While mtDNA encoded proteins barely represent approximately 1% of the mitochondrial proteome, the vast majority of mitochondrial components are encoded in the nuclear genome, synthesized on cytosolic ribosomes and need to be imported into mitochondrial [1]. Mitochondria have an inner and an outer membrane, which segregate mitochondria into two aqueous compartments, the matrix and the intermembrane space (IMS). Therefore, depending on the final destination of the mitochondrial protein inside the organelle, different import pathways take place. In the last years, import machineries and the molecular mechanisms leading to protein translocation inside mitochondria have been studied using different models such as yeast *Saccharomyces cerevisiae*, the fungus *Neurospora crassa* and mammalian cells, therefore being described in detail [2,3,4,5,6,7].

Mutations in components of the OXPHOS system are commonly associated with mitochondrial dysfunction and their link to pathology has been extensively reviewed [8,9,10,11]. Here, we will review the latest molecular details about mitochondrial protein import pathways. In addition, we will discuss new possible candidate genes, which could be responsible of human disorders related to defects in mitochondrial protein translocation. Finally, we will analyse this information and how future perspectives in the field may expand the genetic landscape of mitochondrial diseases.

## 2. Translocation through the Outer Mitochondrial Membrane

The main entry gate to the organelle for almost all mitochondrial precursor proteins is the translocase of the outer membrane (TOM). Three conserved receptor proteins, TOM20, TOM22 and TOM70 (Tom20, Tom22 and Tom70 in yeast), interact with mitochondrial precursors with a different dependency that will vary in accordance with the nature of the incoming protein [12,13,14,15]. TOM40 (Tom40 in yeast) is a β-barrel protein and constitutes the entry channel, forming a pore that was thought to be of approximately 22 Å [16,17,18]. In addition, there are three small TOM proteins, TOM5, TOM6 and TOM7 (Tom5, Tom6 and Tom7 in yeast), which are critical for the stability of the complex [19,20,21]. Interestingly, recent structural analyses in yeast and in human showed that the pore forming subunit Tom40, gathered by the three small TOM proteins and Tom22 can form import-functional dimers or even higher oligomers. With this arrangement, each monomeric unit of the complex contains a single copy of Tom40, Tom22, Tom5, Tom6 and Tom7 and each Tom40 forms a separate pore for protein translocation. In addition, Tom40 barrel was confirmed to consist of 19 β-strands in an antiparallel arrangement (except strands β1 and β19, which are parallel) [22]. Interestingly, the analysis of the human translocase showed that the whole complex has a molecular size of approximately. 150 kDa and the two elliptical pores at the center of the complex formed by TOM40 have an inner cross-sectional diameter of 40 Å by 30 Å, excluding the N-terminal α-helix segment [21]. In addition, every Tom40 channel has two exits for precursors. Presequence containing proteins will be guided inside mitochondria by Tom22, Tom40 and Tom7, whereas intermembrane space (IMS) targeted proteins will be directed with the help of Tom5 and the N-terminal extension of Tom40 [20,21,22,23]. 

The initial translocation reactions that would drive precursors from the mitochondrial surface through the TOM complex are presumably mediated by the high affinity of presequences to negatively-charged and hydrophobic residues present in surface receptors, the Tom40 channel, or IMS domains of import machineries (either TOM or other translocases) [20,24,25,26,27]. Indeed, protein translocation through mitochondrial pores does not happen by simple passive diffusion but *via* specific interactions between pores and precursor proteins. The TOM complex entry pore (formed by the β -barrel subunit Tom40) contains both hydrophilic and hydrophobic regions that allow hydrophilic precursors (like matrix proteins) and hydrophobic ones (for example metabolite carrier proteins) to cross the channel following different paths [3,17,27,28]. Presence of presequences produces a fast gating of the Tom40 channel activity. Interestingly, electrophysiology experiments with purified Tom40 showed that presequences interact and produce gating of the channel, selectively from the cis side [16]. Other studies, using also a reconstituted Tom40 channel, could detect an asymmetrical effect of mitochondrial precursors on channel gating [29]. That may explain unidirectional transport of precursors through the TOM complex. In addition, residence times were calculated using single channel measurements of either recombinant Tom40 or isolated TOM complex from *N*. *crassa* and measuring the average blockage time of the channel when adding mitochondrial presequence peptides. They ranged from 230 to 120 µs, depending on the voltage used [29,30].

**Figure 1:**
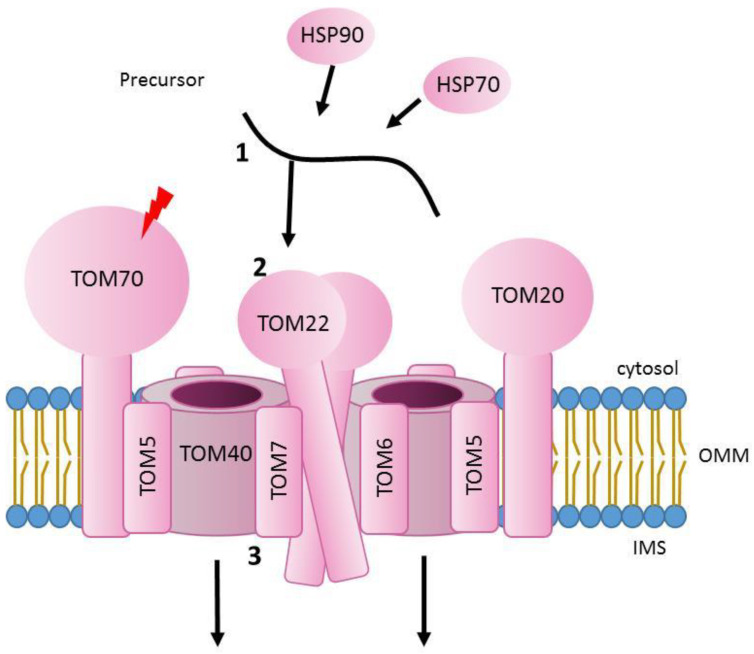
Scheme of translocation of mitochondrial precursors through the outer mitochondrial membrane. 1. Precursors are stabilized in the cytosol with the assistance of specific chaperones like HSP70 and HSP90. 2. Precursors are then recognized by specific receptor subunits (TOM70, TOM22 and TOM20) of TOM complex and directed to the channel forming subunit TOM40. 3. Proteins cross the outer membrane through the channel and continue further to their final destination inside mitochondria. Lightning Bolt: Subunits with known mutations associated with mitochondrial diseases. OMM, outer mitochondrial membrane. IMS, intermembrane space.

Before translocation across the outer membrane happens, proteins have to be transported from cytosolic ribosomes to the TOM complex and both post-translational and co-translocation models have been reported [31,32,33,34,35,36]. In a few reported cases, mRNA of certain proteins are localized to mitochondria and the interaction of nascent peptides with TOM receptors promote recruitment of translating ribosomes to the mitochondrial surface, therefore allowing co-translational import into mitochondria [34,37]. In contrast, proteins that are post-translationally translocated need to be stabilised by cytosolic chaperones like HSP70 and HSP90. These proteins will not only keep mitochondrial precursors in an import-competent state, but will also guide them to the TOM complex [38] (Figure 1). 

## 3. Mutations Affecting TOM Complex

Several patients carrying pathological mutations in the receptor subunit TOM70 (encoded by the gene *TOMM70*) have been described so far. Wei et al. reported a patient presenting with severe anaemia, lactic acidosis, and developmental delay. Two heterozygous compound mutations were found in this patient (p.T265M and p.A582V). Functional characterization of the patient’s lymphocytes showed decreased TOM70 expression and decreased oligomerised TOM70 and TOM22/20/40 complexes, resulting in a multi-OXPHOS deficiency [39]. Dutta et al. additionally described two different patients with TOM70 mutations. The first patient presented with severe global developmental delay, mild acquired microcephaly, hypotonia, mixed hyperkinetic movement disorder (choreoathetosis, dystonia and ataxia), exaggerated startle response and irritability and was found to carry a p.T607I mutation. The second patient (carrying a p.I554F variant) presented among other symptoms with episodic regression starting at four years of age, significant gross motor impairment, and other related complications (Table 1). Complementary studies in a *Drosophila melanogaster* model showed that both patient mutations could rescue to a lesser extent the phenotypes observed in the Tom70 null-mutant allele and Tom70 eye-specific knockdowns when compared to wild type Tom70, indicating that both variants are partial loss-of-function alleles [40].

## 4. Import to the Intermembrane Space Using Disulfide Bridge Formation

**Figure 2:**
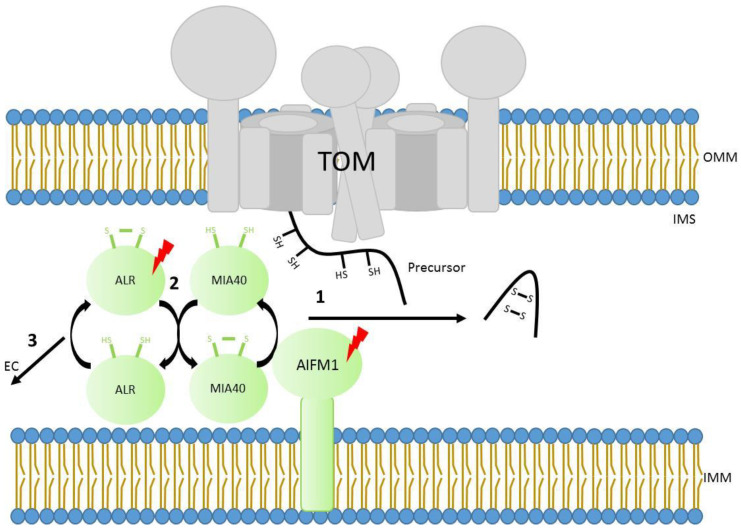
Scheme of translocation and stabilization of mitochondrial precursors residing in the IMS. 1. After crossing the outer membrane through the TOM complex, precursors containing specific cysteine motives are recognized by MIA40 and oxidized, trapping precursors in the IMS. AIFM1 regulates MIA40 mediated import 2. The cysteines in MIA40 are reduced during the trapping of precursors, being re-oxidized by ALR. 3. ALR also regenerates its oxidized cysteines by transferring the electrons to Cyt *c* and molecular oxygen in the electron transport chain (EC). Lightning Bolt: Subunits with known mutations associated with mitochondrial diseases. OMM, outer mitochondrial membrane. IMS, intermembrane space. IMM, inner mitochondrial membrane.

Some mitochondrial proteins reside in the intermembrane space (IMS). Typically, these small soluble proteins contain characteristic twin cysteine motifs, which are ultimately oxidized in order to form disulfide bridges that stabilize them in the compartment. The mitochondrial intermembrane space import and assembly pathway (MIA) works in human mitochondria with the assistance of two main components, MIA40 (also known in human as CHCHD4) and ALR (encoded by the gene *GFER* and homolog of yeast protein Erv1) [114,115,116]. After crossing the TOM40 channel, precursor proteins are recognized by MIA40 which forms transient disulfide bonds with them. Interestingly, cysteine motifs are not the only motifs involved in the recognition, but certain hydrophobic signals are also required for recognition by MIA40 [117]. The disulfide bonds then transfer to the precursors, leading to intramolecular disulfide bridge formation and the stabilization of precursor proteins in the IMS. Cysteine residues present at MIA40 get reduced at every transfer of a disulfide bond to precursor proteins and are subsequently re-oxidized by ALR [115,118,119,120]. Interestingly, human MIA40 has an additional interacting partner (the mitochondrial apoptosis-inducing factor AIF or AIFM1), which modulates the redox regulated MIA40 dependent import into mitochondria. It has been described that AIFM1 interacts with and controls the mitochondrial import of MIA40 itself, therefore altering its function inside the organelle [121,122] (Figure 2). The mitochondrial AIFM1 was the first discovered caspase-independent cell death effector that induces isolated nuclei to adopt apoptotic features [123]. Beyond its role in programmed-cell death signalling pathway, this factor is critical for the proper function of the mitochondrial respiratory chain, specifically respiratory complex I [124]. This observation has been confirmed in different model systems [125,126]. Known MIA40 substrates include factors involved in the biogenesis of respiratory chain complexes such as COX17, COX19, CMC1, and COX6B1 [127]. Indeed, some components of respiratory complex I have been suggested to be also substrates of MIA40 (NDUFS5, NDUFA8, NDUFB7, and NDUFB10) [120,128,129,130]. Therefore, it is plausible that the respiratory chain deficiency with particular complex I distress observed in AIFM1 disease models could be due to a defect of mitochondrial protein targeting to the IMS.

## 5. Mutations in the Mitochondrial Disulfide Relay System

Mutations in MIA40 have not been found to date. However, mutations in ALR have been found associated with an infantile mitochondrial disorder. Three different children presenting with progressive myopathy and partial combined respiratory-chain deficiency, congenital cataract, sensorineural hearing loss, and developmental delay, carried a homozygous mutation in the *GFER* gene (ALR protein) (p.R194H). Molecular analysis performed on the patients’ samples showed reduction of respiratory complexes I, II, and IV activity; abnormal mitochondrial ultrastructure and accelerated time-dependent accumulation of multiple mtDNA deletions. Interestingly, this study additionally found a decreased content of cysteine-rich proteins, which reflects an impaired import of proteins to the IMS [41]. Mutations in ALR have also been associated with adrenal insufficiency, lactic acidosis, congenital cataracts, and respiratory insufficiency secondary to mitochondrial disorder. The 19-year old woman who was originally reported by North et al was found to carry compound heterozygous ALR mutations p.Q125* and p.R194 H. [42,43]. Four additional patients were described by Nambot et al., having similar phenotypes to those previously described (congenital cataracts, progressive muscular hypotonia, hypotrophy, and moderate to severe psychomotor delay). All of them were found to carry heterozygous compound mutations in ALR. Two of them carried a p.C74Afs*76 mutation combined with a C259-25_25924delCA. The other two patients were found to carry the already described p.R194H mutation in combination with a p.A73Pfs*77. Surprisingly, these two last patients also carried a mutant variant in the AGK gene, p.T268N which could also be influencing the phenotype [44] (Table 1). Defects on AGK linked to defective mitochondrial protein import will be discussed later on in this review.

Mutations in the *AIFM1* gene, coding for AIFM1 protein have been associated with different disease conditions during the last decade [131]. A mutation causing severe mitochondrial encephalomyopathy was found in two cousins in homoplasmy. The mutation consisted in a homozygous trinucleotide deletion (c.del601-603) in exon 5 predicting the ablation of an arginine (R) residue at position 201 in AIFM1 protein (p.R201del). OXPHOS activities were reduced in some tissues like fibroblasts or muscle. Interestingly, mtDNA copy number was also reduced to certain extent in both patients [45]. The second mutation identified in the *AIFM1* gene was related to ventriculomegaly at early gestation with decreased activities of respiratory chain complexes I and IV. The mutation was an hemizygous change causing an amino acid change p.G308E [46]. Interestingly, other mutations have been described to alter the redox and pro-apoptotic properties of AIFM1 but not the activities of OXPHOS complexes, like the p.E493V mutation found in a patient with Cowchock syndrome (also known as CMTX4) a recessive disorder with axonal neuropathy, deafness, and cognitive impairment [47]. A different hemizygous mutation (p.M171I) has been identified in a Chinese patient with CMTX4 and mitochondrial abnormalities in nerve fibres and muscle [48]. A slowly progressive mitochondrial encephalomyopathy was described by Ardissone and colleagues to be caused by another hemizygous mutation (p. G262S), presenting also with impaired OXPHOS capacity in muscle sample [49]. Another hemizygous mutation (p. V243L) was identified in a patient with progressive muscular atrophy, ataxia, hearing loss and external opthalmoplegia. Analysis of the patient’s muscle revealed decreased complex I activity [50]. A child with mitochondrial encephalomyopathy and additional complications did not show an altered OXPHOS staining in muscle fibres. Exome sequencing of this patient identified a p.Q479R change in *AIFM1* gene [51]. Two patients presenting with the already described encephalopathy and ventriculomegaly combined with involvement of motor neurons and that showed a deficiency of complex IV activity were found to carry an hemizygous (p. G338E) variant [52]. In addition, a p.F210L change was found in a family with isolated late-onset axonal polyneuropathy and caused a decreased assembly of complex I and III. Interestingly, AIFM1 mutation did not disrupt the interaction with MIA40 [53]. A mutation in the same residue (p.F210S) has been associated with early-onset axonal polyneuropathy and mitochondrial fragmentation [54]. Expanding the spectrum of disorders caused by AIFM1 defects, Zong et al identified eleven new mutations in Chinese patients presenting with auditory neuropathy spectrum disorder (ANSD) with or without peripheral neuropathy (p.T260A, p.G360R, p.R430C, p.V498M, p.I591M, p.A472V p.P475L p.R451Q, p.T260A, p.L344F, p.R422W and p.R422Q). However, there is no information about OXPHOS deficiencies in these patients [55]. In the same way, a new variant (p.D237G) was identified in two unrelated families presenting with spondyloepimetaphyseal dysplasia with mental retardation (SEMD-MR) where no evidence of diminished OXPHOS activities was found [56]. A similar study analysed six different families suffering from the combination of hypomyelinating leukodystrophy and spondylometaphyseal dysplasia (H-SMD) and found different mutations in the *AIFM1* gene, such as the missense changes p.D237G, p.D237V, p.Q235H, the synonymous change p. D240D or a deletion close to a splicing site (c.697-44 T > G). The study proposed all mutations to destabilize either mRNA or AIFM1 protein levels [57]. Recent studies have identified more mutations in the AFM1 gene. Heimer and co-workers identified hemizygous p.M340T and p.T141I variants in two patients with distinct phenotypes, including cerebellar ataxia. Both mutations are in the vicinity of FAD binding site, being plausible that they would affect this interaction. Remarkably, ataxia was partly improved by riboflavin supplementation [58]. Another recent study found a missense variant (p.E453Q) in a Japanese patient with ataxic sensory neuropathy and hearing impairment. In this case, riboflavin supplementation led to no phenotypic improvement [59]. Three members of a family presenting with a complex syndrome (cerebellar ataxia and atrophy, mood and behavioural disorder, intellectual disability with or without hearing loss or peripheral neuropathy), were found to carry the p.G399S mutation in the NADH binding domain of AIFM1 [60]. Finally, a Moroccan family suffering from X-linked auditory neuropathy was found to harbour a missense variant (p.S349G) in the *AIFM1* gene [61] (Table 1). It is interesting to note that apart from the decreased OXPHOS activities identified in some of the patients and although some studies on AIFM1 function have been performed in vitro [45,47,132] or animal models [133,134], there is little evidence of the impact of these variants on the MIA40 function regulation by AIFM1 in these patients. 

## 6. The Presequence Pathway

The majority of mitochondrial precursors (approximately two thirds) have a positively charged presequence located at their N-terminal segment [31]. The presequence consists of approximately 15–20 amino acids that target these precursor proteins to the TOM complex where they are subsequently passed to the translocase of the inner membrane 23 (TIM23 complex). Precursors are directed either to the inner membrane or the matrix and the presequence is finally cleaved by the mitochondrial presequence peptidase [6]. Additional peptidases like the intermediate cleaving peptidase (X-Pro aminopeptidase 3 in human / Icp55 in yeast) and the octapeptidyl peptidase MIP (human homolog of yeast Oct1) further process N-termini of mitochondrial precursors to remove destabilizing amino acids, therefore, reducing degradation by matrix proteases [135,136,137]. Certain chaperones like mtHSP70 and the HSP60–HSP10 chaperonin complex help translocated proteins to fold into their active form [138,139]. In human mitochondria, the core TIM23 complex is formed by the receptor subunit TIM50 (Tim50 in yeast), which has a high affinity to bind precursors through its IMS domain, and the channel forming subunits TIM23 (Tim23 in yeast) and TIM17A/B (Tim17 in yeast) [140,141,142]. This inner membrane channel was defined to form a pore of 13–24 Å in yeast mitochondria [143] and recently structural studies using the same model organism showed it to be 12–21 Å [144]. Further studies in yeast have addressed the dynamics of the translocation process: once the precursor protein has been handed over from the receptor Tim50 to the IMS domain of Tim23, Tim50 leaves the translocase. Until this point, the Tim23 was present as a dimer and in a close conformation. Upon release of Tim50 the dimer dissociates and the channel is activated [145,146]. Once the channel opens, the mitochondrial precursor (which has a positively charged presequence) is translocated through the channel by the membrane potential [147]. Membrane potential is not the only driven import force through Tim23 pore, since the channel is lined with cation selective amino acids that will interact specifically with the positively charged presequence and further promote translocation through the channel [148]. 

As mentioned above, TIM23 is able to either insert precursors in the inner membrane, or to completely translocate them to the matrix. This is possible due to the coexistence of two different forms of the complex, TIM23 ^SORT^, which contains TIM21 (homolog of yeast Tim21) and ROMO1 (also known as ROS modulator 1 and homolog of yeast Mgr2), and the motor associated form (TIM23^MOTOR^), which lacks TIM21 and contains the motor components TIM44, DNAJC15 and DNAJC19 (homologs of yeast Pam18), MAGMAS (or human PAM16), mtHSP70 (or Mortalin) and GrpE [140,142,149,150,151,152,153,154,155].

**Figure 3:**
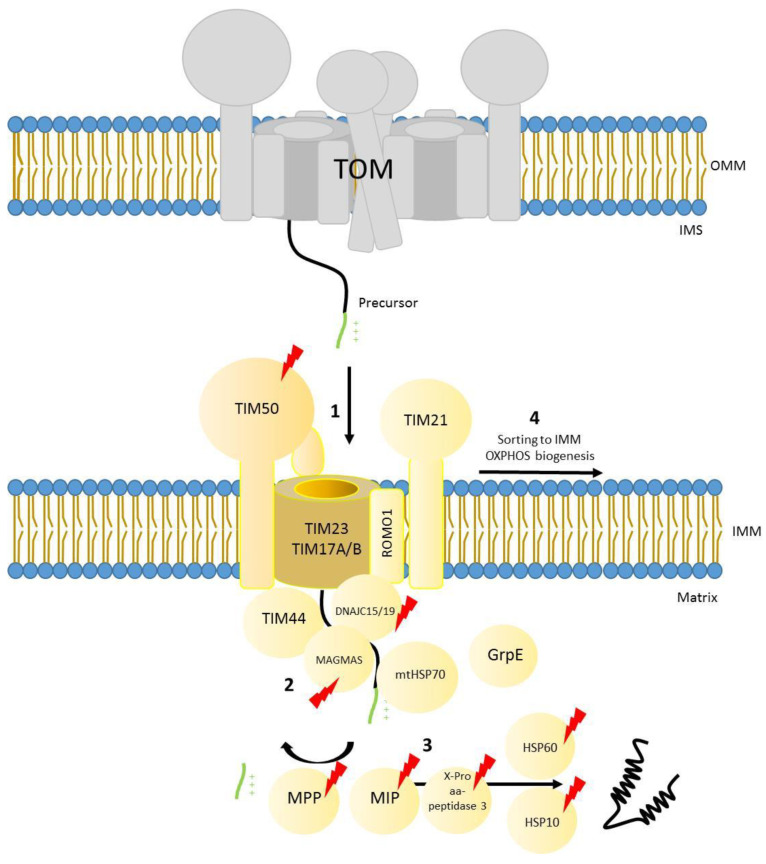
Scheme of translocation of mitochondrial precursors through the inner mitochondrial membrane. 1. After crossing the outer membrane through the TOM complex, presequence containing proteins are recognized by the receptors on the TIM 23 complex and directed to the channel formed by TIM23/TIM17A/B. 2. Proteins targeted to the mitochondrial matrix cross the channel partially, expose their N-terminus to the matrix and interact with TIM23 associated motor components (TIM44, MAGMAS, DNJAC15/19). The chaperone mt-HSP70 translocates the incoming polypeptide completely inside the matrix in consecutive ATP hydrolysis cycles. 3. After translocation to the matrix, presequence is removed by MPP and precursor proteins are further processed by the MIP protease and the X-Pro aminopeptidase 3. Finally, mitochondrial precursors adopt their final conformation with the assistance of the HSP60 and HSP10 complex. 4. Inner membrane proteins are sorted laterally from TIM23 complex into the lipid bilayer. Nuclear-encoded OXPHOS components are ushered by TIM21 into assembly intermediates to converge with mt-encoded subunits in the assembly process. Lightning Bolt: Subunits with known mutations associated with mitochondrial diseases. OMM, outer mitochondrial membrane. IMS, intermembrane space. IMM, inner mitochondrial membrane.

The sorting form of the complex is responsible for the insertion of precursors that contain an extra hydrophobic stop transfer sequence into the lipid bilayer using the membrane potential as driving force. On the other hand, the motor associated form uses the driving force provided by the ATP-activated chaperone mtHSP70, as well as the membrane potential, to translocate precursors into the matrix. The molecular mechanisms leading to protein import into mitochondria through the presequence pathway have been intensively studied in the last decades in the model organism *Saccharomyces cerevisiae* [6] and although the process is generally conserved in human mitochondria, there are some aspects that have evolved to cope with the higher complexity present in mammalian mitochondria. For example, TIM21 shuttles the incoming subunits of complex I and IV from TIM23 to assembly intermediates termed MITRAC (Mitochondrial translation regulation assembly intermediate of cytochrome *c* oxidase), thereby coordinating the translation of mitochondrial encoded subunits with nuclear encoded (imported) ones [153,156,157] (Figure 3). The human translocase is also sensitive to different kinds of cellular insults and is able to adapt to them. Upon stress induction, TIM17A is degraded by the protease YME1L, decreasing the presequence import rate into mitochondria [158]. In addition, TIM23 components are downregulated in hypoxia in a YME1L dependent manner [159]. Interestingly, YME1L import depends on ROMO1 function, therefore, connecting in a reciprocal manner import and proteolytic activity inside mitochondria [155].

## 7. Defects in Presequence Dependent Import

Mutations in the core component of the TIM23 translocase, TIM50, have been recently reported by several groups. Shahrour and colleagues identified two homozygous missense mutations in the *TIMM50* gene (p.R217W and p.T252M) in two patients that presented with intellectual disability and seizure disorder, accompanied by slightly elevated lactate level, 3-methylglutaconic aciduria and variable deficiency of mitochondrial complex V [62]. Reyes and co-workers described a patient with severe epilepsy and lactic acidosis that carried heterozygous compound mutations in TIM50 (p.S112* and p.G190A). Patient fibroblasts showed reduced levels of TIM50 and impaired TIM23 mediated import [63]. Recently, an additional patient was also found to be carrying heterozygous compound mutations in TIM50 (p.R114Q and p.G269S). The diseased individual suffered from visual loss, West syndrome, neutropenia, cardiomyopathy, Leigh syndrome, and persistent 3-methylglutaconic aciduria. Cellular studies confirmed reduced levels of TIM50 and OXPHOS complexes [64] (Table 1).

In addition, mutations in motor components have been associated with mitochondrial disease. A homozygous mutation (p.N76D) in MAGMAS (encoded by the gene *PAM16*) was reported in two families with early lethal spondylodysplastic dysplasia, highlighting the important role of mitochondrial import in ossification [65]. A second mutation was identified in a patient with a milder phenotype and longer survival (p.Q74P) [66]. Mutations in the chaperone DNAJC19 (encoded by the *DNAJC19* gene and homolog of Pam18) have been associated with dilated cardiomyopathy with ataxia (DCMA) (IVS3-1G>C, c.300delA resulting in a p.A100fs*11 frameshift) (see Table 1) [67,68], as well as in combination with progressive cerebellar atrophy (c.280+1_280+5delGTAAG) [69] or with sensorineural hearing loss and bilateral basal ganglia lesions (p.Tyr21*) [70]. Interestingly, DNACJ19 has been shown to interact with prohibitins. Loss of this interaction affects cardiolipin acylation in a similar way as defects in Tafazzin, causative of Barth syndrome. Indeed, the DNAJC19-prohibitin complex may regulate cardiolipin remodeling by Tafazzin, and would explain the similar cardiac phenotype observed in Barth syndrome and DNAJC19 associated disorders [160] (Table 1).

Finally, mutations in either the cleavage of imported precursors or in the chaperones required for the proper active folding after import also lead to disease conditions. Mutations in the *HSPD1* gene, encoding the protein HSP60, have been found in patients with hypomyelinating leukodystrophies like Pelizaeus–Merzbacher disease (p. D29G) [71,72], with hereditary spastic paraplegia (p. V72I) [73] or with familiar dilated cardiomyopathy (p.T320A) [74]. In a similar way, a heterozygous mutation in the *HSPE1* gene ( coding for the protein HSP10) (p.L73F) was found in a patient with neurological and developmental disorder including spasms, hypotonia, developmental delay, and macrocephaly [75]. Similarly, mutations in different subunits of the mitochondrial processing peptidase (MPP) have been associated with different phenotypes. Changes in the α subunit of MPP (encoded by the *PMPCA* gene) have been reported to cause non-progressive cerebellar ataxia (homozygous p.A377T, and compound heterozygous mutations p.S96L and p.G515R) [76], (homozygous p.V256M) [77], or to be responsible for multisystem involvement including profound global developmental delay, severe hypotonia and weakness, respiratory insufficiency, blindness, and lactic acidemia (compound heterozygous p.G356S and p.A377T) [78]. Mutations in the β subunit of MPP (encoded by the *PMPCB* gene) (compound heterozygous p.R175C, and p.A201P, compound heterozygous p.V177G and p.R175H, homozygous p.I422T) have been associated with an early-onset neurodegenerative disorder including symptoms like significant developmental regression, truncal hypotonia, lack of independent ambulation, lack of speech, seizures, ataxia and dystonia. [79]. Interestingly, other factors involved in the further processing of mitochondrial precursors have been linked to disease. Mutations in the *MIPEP* gene, (encoding the octapeptidyl peptidase MIP, also known as Oct1 in yeast) have been found associated with left ventricular non-compaction cardiomyopathy (LVNC), hypotonia and developmental delay. This peptidase is also responsible of shortening N-termini of mitochondrial precursors. The two patients were identified to carry different compound heterozygous mutations (p.L582R, p.L71Q and p. E602*, p.L306F), a third one was carrying an homozygous mutation (p.K343E) and a forth one carried a point mutation (p.H512D) combined with a big deletion in the chromosome where the gene is located [80]. Mutations in the X-Pro aminopeptidase 3, (encoded by the *XPNPEP3* gene) have been associated with severe kidney disorders. One study identified a single heterozygous variant in the *XPNPEP3* gene (p.R155W) associated with severe nephronophthisis associated ciliopathy (NPHP-AC) [81]. A second study identified two new variants (1357G>T and c.931_934 delAACA) in different probands, all of them presenting with different grades of autosomal recessive nephronophthisis [82]. Finally, a recent study identify a new homozygous variant in the *XPNPEP3* gene (p.Q241Tfs*13) associated with a paediatric nephronophthisis [83]. (Table 1).

## 8. Carrier Translocase Mediated Import across the Inner Membrane

**Figure 4:**
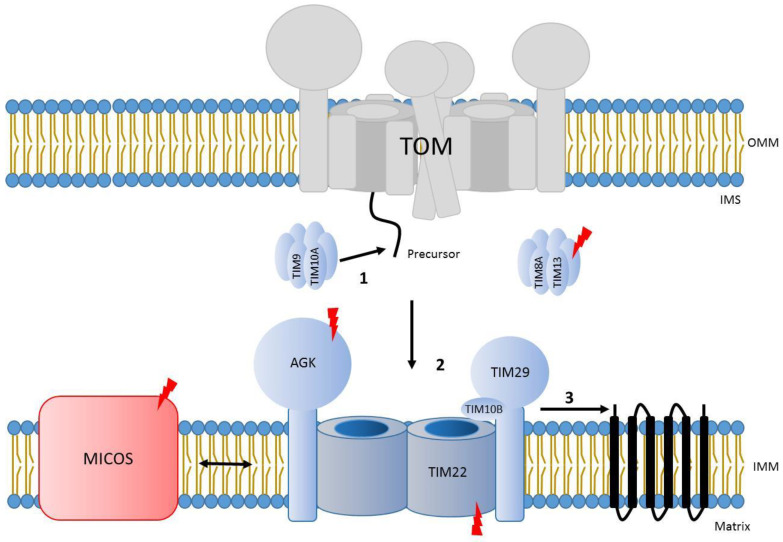
Scheme of insertion of TIM22 substrates in the inner mitochondrial membrane. 1. After crossing the outer membrane through the TOM complex, cargo proteins of the TIM22 translocase are stabilized in the IMS by small TIMs chaperone ring. 2. Small TIMs ring-precursor complex dock to the TIM22 complex and precursors are directed to the twin-pore channel. 3. Precursors are inserted in the lipid bilayer in a sequential mechanism. Interaction with MICOS complex promotes efficient TIM22 mediated import. Lightning Bolt: Subunits with known mutations associated with mitochondrial diseases. OMM, outer mitochondrial membrane. IMS, intermembrane space. IMM, inner mitochondrial membrane.

The carrier translocase (TIM22 complex) facilities the insertion of carrier proteins into the inner membrane. These substrates contain several transmembrane spans in addition to an internal targeting sequence [31,161]. Typically, TIM22 cargo proteins included six transmembrane span-containing carrier proteins and four transmembrane span channel-forming subunits of the TIM23 complex or TIM22 itself [4,162,163,164,165,166,167,168]. Recently, subunits of the mitochondrial pyruvate carrier (MPC) were identified as unconventional TIM22 cargo proteins containing two or three predicted transmembrane spans [169,170]. In addition, sideroflexins (SFXN), which contain five transmembrane segments, were also recently described as cargos of this complex [171]. The human TIM22 complex is a complex of 440-kDa comprised of TIM22, TIM29, TIM10B, and the lipid kinase AGK [165,166,167,168]. Studies in yeast have shown that the translocation of precursors through the TIM22 complex is mediated by two different hexameric rings of small Tim chaperones (Tim9/10 and Tim8/13), which facilitate the transfer of precursors to the TIM22 complex and present certain substrate specificity [172,173,174]. Although these rings are conserved in human mitochondria, only the ring TIM9/10A has been found to be part of the human translocase. It binds to TIM22 cargos upon translocation though the TOM complex, stabilizing them until the ring docks to the complex at multiple binding sites and the precursor is inserted into the inner membrane [165,175,176,177]. On the other hand, the pair TIM8/TIM13 is dispensable for TIM22-mediated import. Indeed, the two human isoforms of TIM8 (TIM8A and B) have been associated to novel complex IV assembly functions in certain cell types [178]. Interestingly, previous evidence described the TIM22 subunit as a central twin-pore forming unit [165,179,180], but recent structural data suggests that a single molecule of TIM22 is present in the complex [176,177]. The TIM22 complex interacts with other structures or complexes within mitochondria in order to modulate protein translocation. The mitochondrial contact site and cristae organization system (MICOS) interacts with the TIM22 complex in human mitochondria to promote efficient import of metabolite carrier proteins, probably by positioning TIM22 at the cristae junction and potentially in the vicinity of the TOM complex [181]. In addition, yeast studies have shown that the voltage-dependent anion channel (VDAC), or porin, interacts with TIM22 substrates in the IMS, therefore recruiting TIM22 to promote efficient transport of these precursors into the inner membrane [182]. Interestingly, porin also participates in the biogenesis of the TOM complex [183] being, therefore, critical for protein translocation into mitochondria (Figure 4).

## 9. Defects in Carrier Transport Associated with Disease

The first identified disease causing mutations related to import through the TIM22 pathway were linked to the Mohr-Tranebjaerg syndrome (MTS), a rare X-linked recessive form of deafness associated with dystonia and other neurological abnormalities [184,185]. The disease causing locus was mapped to the long arm of the X-chromosome and pathological mutations were consequently found in a new gene termed *DDP1* (deafness-dystonia peptide) [84,185]. Homology studies identified DDP1 (also known as TIM8A) as the human homolog of yeast Tim8, which interacts with its counterpart Tim13 to form hexameric rings in the IMS that stabilize hydrophobic precursors before their insertion in the inner membrane [172,186,187,188]. Different mutations in the *DDP*1 gene have been described during the years, including whole gene deletion [84], frameshifts (151delT, 183del10, 108delG and p.C43Vfs*22) [84,85,86,185], stop mutations (p. E24*, p. R80*, p. Q38*, p. Q28*) [87,88,89,90], point mutations (p. C66W, p. M1I) [91,92], intronic mutations (IVS1-23A>C, IVS1+1G>A, IVS1+1G>T) [93,94,95], or micro deletions of the X-chromosome [96]. Indeed, micro deletions of different lengths located in the X-chromosome have been associated with MTS, combined with other pathologies like X-linked agammaglobulinemia (XLA) [97,98,99] (Table 1). 

Mutations in AGK (encoded by the *AGK* gene) were firstly associated with myopathy, combined complex I, III and IV deficiency, bilateral cataracts, and severe mtDNA depletion in skeletal muscle. Two unrelated patients with similar phenotypes carried three different mutations. The first patient harboured a homozygous splicing variant c.1131+1G>T, while the second patient carried two heterozygous mutations, a nonsense variant (p.Y390*) and a splice variant (c.297+2T>C) [100]. Sengers syndrome is a recessive disease characterized by congenital cataracts, hypertrophic cardiomyopathy, skeletal myopathy, exercise intolerance, and lactic acidosis [189]. Mayr et al identified 12 pathogenic alleles with predicted loss of function in the *AGK* gene in Sengers syndrome patients (see Table 1) [101]. Interestingly, and although AGK was not yet described as a component of the TIM22 complex, decreased levels of Adenine nucleotide transporter 1 (a metabolite carrier) were reported for Sengers syndrome patients [101,190]. Additional studies in patients presenting with various forms of this syndrome, identified already described or new mutations in the *AGK* gene (p.M1I, p.K327*, p.I175Yfs*2, c.424-1G>A, p.R137*, p.I346Yfs*39, p.L75Qfs*12, p.R281*, p.Q291*, c.877+3G>T, p.Q291*, p.F406Vfs*4) [102,103,104,105]. Two independent studies characterized AGK as a component of the TIM22 complex and showed that this lipid kinase was required for proper metabolite carrier import into the inner membrane [167,168]. Interestingly, previous reported patients with AGK mutations were shown to have a decreased TIM22 complex and metabolite carrier import into mitochondria [167]. Mutations in AGK have been associated also with milder phenotypes like cataracts. The splicing variant c.424-3C>G resulted in a complete deletion of exon 8 and premature truncation p.A142Tfs*4 (Table 1) [106,191].

To date no pathological mutations have been described for TIM29 or TIM10B and only one disease-linked case has been found in TIM22 (encoded by *TIMM22* gene and homolog of yeast Tim22). A patient presenting with hypotonia, gastroesophageal reflux disease and persistently elevated serum and cerebrospinal fluid lactate was found to carry two heterozygous mutations (p.Y25* and p. V33L) in TIM22. Interestingly, cellular studies showed that the mutation destabilizes the TIM22 complex, as well as levels of cargo proteins [107] (Table 1).

Due to the regulation of TIM22 mediated import through interaction with other complexes inside mitochondria, the spectrum of potential mutations that could indirectly affect the transport of metabolite carriers and other cargos to the inner mitochondrial membrane has expanded. For instance, mutations in subunits of the MICOS complex have been described to be associated with different mitochondrial disorders. Two siblings that presented with severe mitochondrial encephalopathy and recurrent bouts of liver disease were identified to have a homozygous mutation in the *MICOS13* gene (coding for MIC13 protein, also known as QIL1) (c.30-1G>A), resulting in a functionally *null* allele [108]. Additional studies found new mutations in MIC13 associated with mitochondrial encephalopathy (c.13_29del causing the frameshift p.W6Pfs*71, c.44delC causing the frameshift p.G15Efs*75, c.260-2A>G causing two aberrant splicing sites) [109,110,111]. A mutation in the gene *APOO*, encoding for MIC26 (p.I117T), was recently identified in a patient with a complex phenotype (including progressive developmental delay, lactic acidosis, muscle weakness, hypotonia, weight loss, gastrointestinal and body temperature dysautonomia, repetitive infections, cognitive impairment and autistic behaviour [112] (Table 1). Despite these findings, further analyses should be performed to clarify the impact of MICOS patient mutations on the translocation of proteins to the inner mitochondrial membrane through the TIM22 complex.

## 10. Other Import Pathways

β-barrel proteins located in the outer mitochondrial membrane fulfil critical roles in the transport of metabolites or other proteins inside mitochondria. Well-known β-barrel proteins are the different isoforms of VDAC or the channel forming subunit of the TOM complex TOM40 [16,192]. These precursors are stabilized by cytosolic chaperones and once they get recognised by TOM receptors, they are translocated inside mitochondria, passing TOM40 channel and being transferred to the SAM (sorting and assembly machinery complex) [192,193,194]. Since TOM and SAM complexes are linked to guarantee efficient substrate channelling, pathologic defects on TOM complex (described above) would indirectly affect insertion of β-barrel proteins through SAM complex. Very recently, patients carrying mutations in the *MTX2* gene, coding for the proposed human homolog of the yeast Sam35 (core component of SAM complex) [195] were described in several patients presenting with Mandibuloacral dysplasia (MAD), a progeroid disorder commonly associated with defects of the nuclear lamina. This study identified five different *null* variants (c.2T>A leading to p.M1L, c.544-1G>C leading to a p.V182Rfs*3 frameshift, c.208+3_208+6del leading to a p.A46Vfs*12 frameshift, c.603del leading to p.Y202Ifs*26 and c.294_295delT leading to p.L99*) (Table 1). The mutants decreased the levels of MTX2 and MTX1 (proposed human homolog of Sam37) [113]. However, although these two proteins have been predicted to share some homology with the yeast counterparts of SAM machinery, and required for proper VDAC and TOM40 import, they were observed in a different complex than SAM50. Therefore, their exact role in the insertion of β-barrel proteins to the outer membrane remains unclear [195]. Interestingly, studies in model organisms showed that SAM complex interacts with elements of the MICOS complex. Deletion of core MICOS components impacts β-barrel protein insertion [196]. Therefore, it is tempting to speculate that pathologic mutations in this inner membrane complex (revised above) would also affect the insertion of proteins in the outer mitochondrial membrane. Finally, small Tims chaperones are known to accompany precursors from TOM to SAM complex in yeast mitochondria [174]. Although pathologic mutations have been identified in TIM8 (see above), the role of TIM8/13 on protein translocation in human mitochondria is still under debate [178]. 

Outer membrane proteins with an α-helical structure are inserted without the assistance of TOM40. That includes single-spanning proteins with either an amino-terminal membrane anchor or a carboxy-terminal anchor and finally multi-spanning proteins. They are translocated through the mitochondrial import channel for membrane insertion (MIM) [197]. Studies in yeast mitochondria revealed that in some cases, the process is assisted by Tom70 [198,199]. In others the lipid composition seems to be critical for protein insertion, although the exact molecular mechanism remains unknown [200,201]. In any case, no pathological mutations have been identified among components of this particular route.

## 11. Expanding the Genetic Landscape of Mitochondrial Protein Import Disorders

The enhanced understanding of the molecular mechanisms that lead to mitochondrial protein import, achieved in the last years, has significantly improved our comprehension of the disorders derived from them. The discovery of new players involved in these processes, like TIM29, AGK or AIFM1, has increased the number of candidate genes responsible for mitochondrial protein import diseases [121,122,165,166,167,168]. On the other hand, the discovery of the specialization and diversification of human import proteins compared to their yeast counterparts will help to identify specific phenotypes caused by mutations in these proteins. For example, due to the role of ROMO1 in YME1L processing, mutations in ROMO1 may affect not only TIM23 associated import, but also protein quality control and mitochondrial morphology [155]. In the same way, the new role of TIM8A and B in complex IV assembly in certain tissues may also help to explain the pathogenicity of MTS [178].

In addition, other entities inside mitochondria play a regulatory role in protein translocation processes, therefore being susceptible to causing protein import pathologies [181,182,183,202,203]. For example, mutations in MICOS or VDAC components could also affect mitochondrial protein import and cause disease. However, one has to remain cautious since these factors have other additional functions. In-depth molecular studies are, therefore, required to dissect the effect of new mutations on protein translocation pathways. 

Finally, defects in protein translocation into mitochondria have not only been linked to mitochondrial diseases but also with more common diseases like neurodegenerative diseases or cancer [204]. Thus, mitochondrial import routes have been related to different cellular pathways, connections that may be worthy to investigate in order to expand the genetic landscape of these diseases. 

## Figures and Tables

**Table 1:** Mutations identified in different genes involved in protein traslocation and associated with disease.

Mutation	Protein	Import Pathway	Phenotype	Reference
p.T265M p.A582V	TOM70	TOM	anemia, lactic acidosis, and developmental delay	[39]
p.T607I	TOM70	TOM	severe global developmental delay, mild acquired microcephaly, hypotonia, mixed hyperkinetic movement disorder, exaggerated startle response, and irritability	[40]
p.I554F	TOM70	TOM	gross motor impairment, proximal weaknes, spastic ataxia, hypotonia, cogwheeling, truncal titubation, dysmetric motor control, dysarthria, and ptosis	[40]
p.R194H	ALR	MIA40	progressive myopathy and partial combined respiratory-chain deficiency, congenital cataract, sensorineural hearing loss, and developmental delay	[41]
p.Q125* p.R194H	ALR	MIA40	adrenal insufficiency, lactic acidosis, congenital cataracts and respiratory insufficiency secondary to mitochondrial disorder	[42,43]
p.C74Afs*76 C259-25_25924delCA p.R194H p.A73Pfs*77	ALR	MIA40	progressive muscular hypotonia, congenital cataracts, hypotrophy, and moderate to severe psychomotor delay	[44]
p.R201del	AIFM1	MIA40	mitochondrial encephalomyopathy	[45]
p.G308E	AIFM1	MIA40	ventriculomegaly at early gestation	[46]
p.E493V	AIFM1	MIA40	Cowchock syndrome ( CMTX4)	[47]
p.M171I	AIFM1	MIA40	Cowchock syndrome ( CMTX4)	[48]
p.G262S	AIFM1	MIA40	progressive mitochondrial encephalomyopathy	[49]
p.V243L	AIFM1	MIA40	progressive muscular atrophy, ataxia, hearing loss, and external opthalmoplegia	[50]
p.Q479R	AIFM1	MIA40	mitochondrial encephalomyopathy, additional complications	[51]
p.G338E	AIFM1	MIA40	encephalopathy and ventriculomegaly combined with involvement of motor neurons	[52]
p.F210L	AIFM1	MIA40	isolated late onset axonal polyneuropathy	[53]
p.F210S	AIFM1	MIA40	early-onset axonal polyneuropathy	[54]
p.T260A p.G360R p.R430C p.V498M p.I591M p.A472V p.P475L p.R451Q p.T260A p.L344F p.R422W p.R422Q	AIFM1	MIA40	auditory neuropathy spectrum disorder (ANSD) with or without peripheral neuropathy	[55]
p.D237G	AIFM1	MIA40	spondyloepimetaphyseal dysplasia with mental retardation (SEMD-MR)	[56]
p.D237G p.D237V p.Q235H p.D240D c.697-44 T>G (splicing variant)	AIFM1	MIA40	hypomyelinating leukodystrophy and spondylometaphyseal dysplasia (H-SMD)	[57]
p.M340T p.T141I	AIFM1	MIA40	cerebellar ataxia and others	[58]
p.E453Q	AIFM1	MIA40	ataxic sensory neuropathy and hearing impairment	[59]
p.G399S	AIFM1	MIA40	cerebellar ataxia and atrophy, mood and behavioural disorder, intellectual disability with or without hearing loss or peripheral neuropathy	[60]
p.S349G	AIFM1	MIA40	X-linked auditory neuropathy	[61]
p.R217W p.T252M	TIM50	TIM23	intellectual disability and seizure disorder	[62]
p.S112* p.G190A	TIM50	TIM23	severe epilepsy and lactic acidosis	[63]
p.R114Q p.G269S	TIM50	TIM23	visual loss, West syndrome, neutropenia, cardiomyopathy, Leigh syndrome and persistent 3-methylglutaconic aciduria	[64]
p.N76D	MAGMAS	TIM23	early lethal spondylodysplastic dysplasia	[65]
p.Q74P	MAGMAS	TIM23	spondylodysplastic dysplasia	[66]
IVS3-1G>C	DNAJC19	TIM23	dilated cardiomyopathy with ataxia (DCMA)	[67]
p.A100fs*11	DNAJC19	TIM23	dilated cardiomyopathy with ataxia (DCMA)	[68]
c.280+1_280+5delGTAAG	DNAJC19	TIM23	DCMA combined with progressive cerebellar atrophy	[69]
p.Y21*	DNAJC19	TIM23	DCMA with sensorineural hearing loss, bilateral basal ganglia lesions	[70]
p.D29G	HPS60	TIM23	Pelizaeus–Merzbacher disease	[71,72]
p.V72I	HPS60	TIM23	hereditary spastic paraplegia	[73]
p.T320A	HPS60	TIM23	familiar dilated cardiomyopathy	[74]
p.L73F	HSP10	TIM23	neurological and developmental disorder:spasms, hypotonia, developmental delay and macrocephaly	[75]
p.A377T p.S96L p.G515R	α-MPP	TIM23	non-progressive cerebellar ataxia	[76]
p.V256M	α-MPP	TIM23	non-progressive cerebellar ataxia	[77]
p.G356S p.A377T	α-MPP	TIM23	multisystem involvement including profound global developmental delay, severe hypotonia and weakness, respiratory insufficiency, blindness	[78]
p.R175C p.A201P p.V177G p.R175H p.I422T	β-MPP	TIM23	early-onset neurodegenerative disorder:developmental regression, truncal hypotonia, lack of independent ambulation, lack of speech, seizures, ataxia, dystonia	[79]
p.L582R p.L71Q p.E602* p.L306F p.K343E p.H512D macro deletion	MIP	TIM23	left ventricular non-compaction cardiomyopathy, hypotonia, developmental delay	[80]
p.R155W	X-Pro aminopeptidase 3	TIM23	nephronophthisis associated ciliopathy	[81]
1357G>T c.931_934 delAACA	X-Pro aminopeptidase 3	TIM23	kidney disease nephronophthisis	[82]
p.Q241Tfs*13	X-Pro aminopeptidase 3	TIM23	early-age nephronophthisis	[83]
gene deletion 151delT 183del10	TIM8A	TIM22	Mohr-Tranebjaerg syndrome	[84]
108delG	TIM8A	TIM22	Mohr–Tranebjaerg syndrome	[85]
p.C43Vfs*22	TIM8A	TIM22	Mohr–Tranebjaerg syndrome	[86]
p.E24*	TIM8A	TIM22	Mohr–Tranebjaerg syndrome	[87]
p.R80*	TIM8A	TIM22	Mohr–Tranebjaerg syndrome	[88]
p.Q38*	TIM8A	TIM22	Mohr–Tranebjaerg syndrome	[89]
p.Q28*	TIM8A	TIM22	Mohr–Tranebjaerg syndrome	[90]
p.C66W	TIM8A	TIM22	Mohr–Tranebjaerg syndrome	[91]
p.M1I	TIM8A	TIM22	Mohr–Tranebjaerg syndrome	[92]
IVS1-23A>C	TIM8A	TIM22	Mohr–Tranebjaerg syndrome	[93]
IVS1+1G>A	TIM8A	TIM22	Mohr–Tranebjaerg syndrome	[94]
IVS1+1G>T	TIM8A	TIM22	Mohr–Tranebjaerg syndrome	[95]
X-chromosome micro deletions	TIM8A	TIM22	Mohr–Tranebjaerg syndrome	[96]
X-chromosome micro deletions	TIMA8	TIM22	Mohr–Tranebjaerg syndrome and X-linked agammaglobulinemia	[97,98,99]
c.1131+1G>T p.Y390* c.297 + 2T>C	AGK	TIM22	myopathy, bilateral cataracts	[100]
p.Y102∗ p.R281∗ p.M1I p.Q173∗ p.R138∗ p.G380Lfs∗16 p.M1I p.Y224∗ c.1131 + 5G>A p. Q291Rfs∗8 c.101+?_222-?del c.221+1G>A p.Q405∗	AGK	TIM22	Sengers syndrome	[101]
p.M1I p.K327*	AGK	TIM22	Sengers syndrome	[102]
p.I175Yfs*2 c.424-1G>A p.R137* p.Q291* p.I346Yfs*39 p.L75Qfs*12 p. R281* c.877+3G>T	AGK	TIM22	Sengers syndrome	[103]
p.I346Yfs*39	AGK	TIM22	Sengers syndrome	[104]
p.F406Vfs*4	AGK	TIM22	Sengers syndrome	[105]
Exon 8 splicing variant (p.A142Tfs*4)	AGK	TIM22	cataracts	[106]
p.Y25* p.V33L	TIM22	TIM22	hypotonia, gastroesophageal reflux disease, elevated lactate	[107]
c.30-1G>A (splicing variant)	MIC13	TIM22	severe mitochondrial encephalopathy, recurrent bouts of liver disease	[108]
p.G15Efs*75	MIC13	TIM22	mitochondrial encephalopathy	[109]
p.W6Pfs*71	MIC13	TIM22	mitochondrial encephalopathy	[110]
c.260-2A>G	MIC13	TIM22	mitochondrial encephalopathy	[111]
p.I117T	MIC26	TIM22	progressive developmental delay, lactic acidosis, muscle weakness, hypotonia, weight loss, gastrointestinal and body temperature dysautonomia, repetitive infections, cognitive impairment, autistic behaviour	[112]
p.M1L c.544-1G>C splicing variant (p.V182Rfs*3 ) c.208+3_208 + 6del splicing variant(p.A46Vfs*12) p.Y202Ifs*26 p.L99*	MTX2	SAM	Mandibuloacral dysplasia	[113]

Complete protein names: TOM70–Mitochondrial import receptor subunit TOM70. ALR–FAD-linked sulfhydryl oxidase ALR. AIFM1–Apoptosis-inducing factor 1, mitochondrial. TIM50–Mitochondrial import inner membrane translocase subunit TIM50. MAGMAS–Mitochondria-associated granulocyte macrophage CSF-signaling molecule. DNAJC19–Mitochondrial import inner membrane translocase subunit TIM14. HSP60–60 kDa heat shock protein, mitochondrial. HSP10–10 kDa heat shock protein, mitochondrial. α-MPP–Mitochondrial-processing peptidase subunit alpha. β-MPP–Mitochondrial-processing peptidase subunit beta. MIP–Mitochondrial intermediate peptidase. X-Pro aminopeptidase 3–Xaa- Pro aminopeptidase 3. TIM8A–Mitochondrial import inner membrane translocase subunit Tim8 A. AGK–Acylglycerol kinase, mitochondrial. TIM22–Mitochondrial import inner membrane translocase subunit Tim22. MIC13–MICOS complex subunit MIC13. MIC26–MICOS complex subunit MIC26. MTX2–Metaxin-2.

## References

[1] Morgenstern M., Stiller S.B., Lübbert P., Peikert C.D., Dannenmaier S., Drepper F., Weill U., Hoess P., Feuerstein R., Gebert M. (2017). Definition of a High-Confidence Mitochondrial Proteome at Quantitative Scale. Cell Rep..

[2] Neupert W., Herrmann J.M. (2007). Translocation of Proteins into Mitochondria. Annu. Rev. Biochem..

[3] Wiedemann N., Pfanner N. (2017). Mitochondrial Machineries for Protein Import and Assembly. Annu. Rev. Biochem..

[4] Kang Y., Fielden L.F., Stojanovski D. (2018). Mitochondrial protein transport in health and disease. Semin. Cell Dev. Biol..

[5] Pfanner N., Warscheid B., Wiedemann N. (2019). Mitochondrial proteins: From biogenesis to functional networks. Nat. Rev. Mol. Cell Biol..

[6] Callegari S., Cruz-Zaragoza L.D., Rehling P. (2020). From TOM to the TIM23 complex—Handing over of a precursor. Biol. Chem..

[7] Priesnitz C., Pfanner N., Becker T. (2020). Studying protein import into mitochondria. Methods Cell Biol..

[8] McCormick E.M., Zolkipli-Cunningham Z., Falk M.J. (2018). Mitochondrial disease genetics update. Curr. Opin. Pediatr..

[9] Hock D.H., Robinson D.R.L., Stroud D.A. (2020). Blackout in the powerhouse: Clinical phenotypes associated with defects in the assembly of OXPHOS complexes and the mitoribosome. Biochem. J..

[10] Fernandez-Vizarra E., Zeviani M. (2021). Mitochondrial disorders of the OXPHOS system. FEBS Lett..

[11] Ferrari A., Del’Olio S., Barrientos A. (2021). The Diseased Mitoribosome. FEBS Lett..

[12] Van Wilpe S., Ryan M.T., Hill K., Maarse A.C., Meisinger C., Brix J., Dekker P.J.T., Moczko M., Wagner R., Meijer M. (1999). Tom22 is a multifunctional organizer of the mitochondrial preprotein translocase. Nat. Cell Biol..

[13] Abe Y., Shodai T., Muto T., Mihara K., Torii H., Nishikawa S.-I., Endo T., Kohda D. (2000). Structural Basis of Presequence Recognition by the Mitochondrial Protein Import Receptor Tom20. Cell.

[14] Yamamoto H., Fukui K., Takahashi H., Kitamura S., Shiota T., Terao K., Uchida M., Esaki M., Nishikawa S.-I., Yoshihisa T. (2009). Roles of Tom70 in Import of Presequence-containing Mitochondrial Proteins. J. Biol. Chem..

[15] Backes S., Hess S., Boos F., Woellhaf M.W., Gödel S., Jung M., Mühlhaus T., Herrmann J.M. (2018). Tom70 enhances mitochondrial preprotein import efficiency by binding to internal targeting sequences. J. Cell Biol..

[16] Hill K., Model K., Ryan M.T., Dietmeier K., Martin F., Wagner R., Pfanner N. (1998). Tom40 forms the hydrophilic channel of the mitochondrial import pore for preproteins. Nat. Cell Biol..

[17] Melin J., Schulz C., Wrobel L., Bernhard O., Chacinska A., Jahn O., Schmidt B., Rehling P. (2014). Presequence Recognition by the Tom40 Channel Contributes to Precursor Translocation into the Mitochondrial Matrix. Mol. Cell. Biol..

[18] Kuszak A., Jacobs D., Gurnev P.A., Shiota T., Louis J.M., Lithgow T., Bezrukov S.M., Rostovtseva T.K., Buchanan S.K. (2015). Evidence of Distinct Channel Conformations and Substrate Binding Affinities for the Mitochondrial Outer Membrane Protein Translocase Pore Tom40. J. Biol. Chem..

[19] Kato H., Mihara K. (2008). Identification of Tom5 and Tom6 in the preprotein translocase complex of human mitochondrial outer membrane. Biochem. Biophys. Res. Commun..

[20] Bausewein T., Mills D.J., Langer J.D., Nitschke B., Nussberger S., Kühlbrandt W. (2017). Cryo-EM Structure of the TOM Core Complex from Neurospora crassa. Cell.

[21] Wang W., Chen X., Zhang L., Yi J., Ma Q., Yin J., Zhuo W., Gu J., Yang M. (2020). Atomic structure of human TOM core complex. Cell Discov..

[22] Tucker K., Park E. (2019). Cryo-EM structure of the mitochondrial protein-import channel TOM complex at near-atomic resolution. Nat. Struct. Mol. Biol..

[23] Araiso Y., Tsutsumi A., Qiu J., Imai K., Shiota T., Song J., Lindau C., Wenz L.-S., Sakaue H., Yunoki K. (2019). Structure of the mitochondrial import gate reveals distinct preprotein paths. Nat. Cell Biol..

[24] Bolliger L., Junne T., Schatz G., Lithgow T. (1995). Acidic receptor domains on both sides of the outer membrane mediate translocation of precursor proteins into yeast mitochondria. EMBO J..

[25] Kanamori T., Nishikawa S.-I., Shin I., Schultz P.G., Endo T. (1997). Probing the environment along the protein import pathways in yeast mitochondria by site-specific photocrosslinking. Proc. Natl. Acad. Sci. USA.

[26] Mokranjac D., Sichting M., Popov-Čeleketić D., Mapa K., Gevorkyan-Airapetov L., Zohary K., Hell K., Azem A., Neupert W. (2009). Role of Tim50 in the Transfer of Precursor Proteins from the Outer to the Inner Membrane of Mitochondria. Mol. Biol. Cell.

[27] Shiota T., Imai K., Qiu J., Hewitt V., Tan K., Shen H.-H., Sakiyama N., Fukasawa Y., Hayat S., Kamiya M. (2015). Molecular architecture of the active mitochondrial protein gate. Science.

[28] Esaki M., Kanamori T., Nishikawa S.-I., Shin I., Schultz P.G., Endo T. (2003). Tom40 protein import channel binds to non-native proteins and prevents their aggregation. Nat. Struct. Mol. Biol..

[29] Mahendran K.R., Romero-Ruiz M., Schlösinger A., Winterhalter M., Nussberger S. (2012). Protein Translocation through Tom40: Kinetics of Peptide Release. Biophys. J..

[30] Romero-Ruiz M., Mahendran K.R., Eckert R., Winterhalter M., Nussberger S. (2010). Interactions of Mitochondrial Presequence Peptides with the Mitochondrial Outer Membrane Preprotein Translocase TOM. Biophys. J..

[31] Chacinska A., Koehler C.M., Milenkovic D., Lithgow T., Pfanner N. (2009). Importing Mitochondrial Proteins: Machineries and Mechanisms. Cell.

[32] Gadir N., Haim-Vilmovsky L., Kraut-Cohen J., Gerst J.E. (2011). Localization of mRNAs coding for mitochondrial proteins in the yeast Saccharomyces cerevisiae. RNA.

[33] Williams C.C., Jan C.H., Weissman J.S. (2014). Targeting and plasticity of mitochondrial proteins revealed by proximity-specific ribosome profiling. Science.

[34] Gold V.A., Chroscicki P., Bragoszewski P., Chacinska A. (2017). Visualization of cytosolic ribosomes on the surface of mitochondria by electron cryo-tomography. EMBO Rep..

[35] Lesnik C., Cohen Y., Atir-Lande A., Schuldiner M., Arava Y. (2014). OM14 is a mitochondrial receptor for cytosolic ribosomes that supports co-translational import into mitochondria. Nat. Commun..

[36] Hansen K.G., Herrmann J.M. (2019). Transport of Proteins into Mitochondria. Protein J..

[37] Eliyahu E., Pnueli L., Melamed D., Scherrer T., Gerber A.P., Pines O., Rapaport D., Arava Y. (2010). Tom20 mediates localization of mRNAs to mitochondria in a translation-dependent manner. Mol. Cell Biol..

[38] Young J.C., Hoogenraad N.J., Hartl F. (2003). Molecular Chaperones Hsp90 and Hsp70 Deliver Preproteins to the Mitochondrial Import Receptor Tom70. Cell.

[39] Wei X., Du M., Xie J., Luo T., Zhou Y., Zhang K., Li J., Chen D., Xu P., Jia M. (2020). Mutations in TOMM70 lead to multi-OXPHOS deficiencies and cause severe anemia, lactic acidosis, and developmental delay. J. Hum. Genet..

[40] Dutta D., Briere L.C., Kanca O., Marcogliese P.C., Walker M.A., High F.A., Vanderver A., Krier J., Carmichael N., Callahan C. (2020). De novo mutations in TOMM70, a receptor of the mitochondrial import translocase, cause neurological impairment. Hum. Mol. Genet..

[41] Di Fonzo A., Ronchi D., Lodi T., Fassone E., Tigano M., Lamperti C., Corti S., Bordoni A., Fortunato F., Nizzardo M. (2009). The Mitochondrial Disulfide Relay System Protein GFER Is Mutated in Autosomal-Recessive Myopathy with Cataract and Combined Respiratory-Chain Deficiency. Am. J. Hum. Genet..

[42] Calderwood L., Holm I.A., Teot L.A., Anselm I. (2016). Adrenal Insufficiency in Mitochondrial Disease. J. Child. Neurol..

[43] North K., Korson M.S., Krawiecki N., Shoffner J.M., Holm I.A. (1996). Oxidative phosphorylation defect associated with primary adrenal insufficiency. J. Pediatr..

[44] Nambot S., Gavrilov D., Thevenon J., Bruel A., Bainbridge M., Rio M., Goizet C., Rotig A., Jaeken J., Niu N. (2017). Further delineation of a rare recessive encephalomyopathy linked to mutations in GFER thanks to data sharing of whole exome sequencing data. Clin. Genet..

[45] Ghezzi D., Sevrioukova I., Invernizzi F., Lamperti C., Mora M., D’Adamo P., Novara F., Zuffardi O., Uziel G., Zeviani M. (2010). Severe X-Linked Mitochondrial Encephalomyopathy Associated with a Mutation in Apoptosis-Inducing Factor. Am. J. Hum. Genet..

[46] Berger I., Ben-Neriah Z., Dor-Wolman T., Shaag A., Saada A., Zenvirt S., Raas-Rothschild A., Nadjari M., Kaestner K.H., Elpeleg O. (2011). Early prenatal ventriculomegaly due to an AIFM1 mutation identified by linkage analysis and whole exome sequencing. Mol. Genet. Metab..

[47] Rinaldi C., Grunseich C., Sevrioukova I.F., Schindler A., Horkayne-Szakaly I., Lamperti C., Landouré G., Kennerson M.L., Burnett B.G., Bönnemann C. (2012). Cowchock Syndrome Is Associated with a Mutation in Apoptosis-Inducing Factor. Am. J. Hum. Genet..

[48] Wang B., Li X., Wang J., Liu L., Xie Y., Huang S., Pakhrin P.S., Jin Q., Zhu C., Tang B. (2018). A novel AIFM1 mutation in a Chinese family with X-linked Charcot–Marie–Tooth disease type 4. Neuromuscul. Disord..

[49] Ardissone A., Piscosquito G., Legati A., Langella T., Lamantea E., Garavaglia B., Salsano E., Farina L., Moroni I., Pareyson D. (2015). A slowly progressive mitochondrial encephalomyopathy widens the spectrum of AIFM1 disorders. Neurol..

[50] Kettwig M., Schubach M., Zimmermann F.A., Klinge L., Mayr J., Biskup S., Sperl W., Gärtner J., Huppke P. (2015). From ventriculomegaly to severe muscular atrophy: Expansion of the clinical spectrum related to mutations in AIFM1. Mitochondrion.

[51] Morton S.U., Prabhu S.P., Lidov H.G., Shi J., Anselm I., Brownstein C.A., Bainbridge M.N., Beggs A., Vargas S.O., Agrawal P.B. (2017). AIFM1 mutation presenting with fatal encephalomyopathy and mitochondrial disease in an infant. Mol. Case Stud..

[52] Diodato D., Tasca G., Verrigni D., D’Amico A., Rizza T., Tozzi G., Martinelli D., Verardo M., Invernizzi F., Nasca A. (2016). A novel AIFM1 mutation expands the phenotype to an infantile motor neuron disease. Eur. J. Hum. Genet..

[53] Hu B., Wang M., Castoro R., Simmons M., Dortch R., Yawn R., Li J. (2017). A novel missense mutation in AIFM1 results in axonal polyneuropathy and misassembly of OXPHOS complexes. Eur. J. Neurol..

[54] Sancho P., Sánchez-Monteagudo A., Collado A., Marco-Marín C., Domínguez-González C., Camacho A., Knecht E., Espinós C., Lupo V. (2017). A newly distal hereditary motor neuropathy caused by a rare AIFM1 mutation. Neurogenetics.

[55] Zong L., Guan J., Ealy M., Zhang Q., Wang D., Wang H., Zhao Y., Sheng Z., Campbell C.A., Wang F. (2015). Mutations in apoptosis-inducing factor cause X-linked recessive auditory neuropathy spectrum disorder. J. Med. Genet..

[56] Mierzewska H., Rydzanicz M., Biegański T., Kosinska J., Mierzewska-Schmidt M., Ługowska A., Pollak A., Stawiński P., Walczak A., Kędra A. (2017). Spondyloepimetaphyseal dysplasia with neurodegeneration associated withAIFM1mutation—A novel phenotype of the mitochondrial disease. Clin. Genet..

[57] Miyake N., Wolf N.I., Cayami F.K., Crawford J., Bley A., Bulas D., Conant A., Bent S.J., Gripp K.W., Hahn A. (2017). X-linked hypomyelination with spondylometaphyseal dysplasia (H-SMD) associated with mutations in AIFM1. Neurogenetics.

[58] Heimer G., Eyal E., Zhu X., Ruzzo E., Marek-Yagel D., Sagiv D., Anikster Y., Reznik-Wolf H., Pras E., Levi D.O. (2018). Mutations in AIFM1 cause an X-linked childhood cerebellar ataxia partially responsive to riboflavin. Eur. J. Paediatr. Neurol..

[59] Kawarai T., Yamazaki H., Yamakami K., Tsukamoto-Miyashiro A., Kodama M., Rumore R., Caltagirone C., Nishino I., Orlacchio A. (2020). A novel AIFM1 missense mutation in a Japanese patient with ataxic sensory neuronopathy and hearing impairment. J. Neurol. Sci..

[60] Pandolfo M., Rai M., Remiche G., Desmyter L., VanderNoot I. (2020). Cerebellar ataxia, neuropathy, hearing loss, and intellectual disability due to AIFM1 mutation. Neurol. Genet..

[61] Elrharchi S., Riahi Z., Salime S., Charoute H., Elkhattabi L., Boulouiz R., Kabine M., Bonnet C., Petit C., Barakat A. (2021). Novel Mutation in AIFM1 Gene Associated with X-Linked Deafness in a Moroccan Family. Hum. Hered..

[62] Shahrour M., Staretz-Chacham O., Dayan D., Stephen J., Weech A., Damseh N., Chen H.P., Edvardson S., Mazaheri S., Saada A. (2016). Mitochondrial epileptic encephalopathy, 3-methylglutaconic aciduria and variable complex V deficiency associated with TIMM50 mutations. Clin. Genet..

[63] Reyes A., Melchionda L., Burlina A., Robinson A.J., Ghezzi D., Zeviani M. (2018). Mutations in TIMM50 compromise cell survival in OxPhos-dependent metabolic conditions. EMBO Mol. Med..

[64] Tort F., Ugarteburu O., Texidó L., Gea-Sorlí S., García-Villoria J., Ferrer-Cortès X., Arias Á., Matalonga L., Gort L., Ferrer I. (2019). Mutations in TIMM50 cause severe mitochondrial dysfunction by targeting key aspects of mitochondrial physiology. Hum. Mutat..

[65] Mehawej C., Delahodde A., Legeai-Mallet L., Delague V., Kaci N., Desvignes J.-P., Kibar Z., Capo-Chichi J.-M., Chouery E., Munnich A. (2014). The Impairment of MAGMAS Function in Human Is Responsible for a Severe Skeletal Dysplasia. PLoS Genet..

[66] Moosa S., Fano V., Obregon M.G., Altmüller J., Thiele H., Nürnberg P., Nishimura G., Wollnik B. (2016). A novel homozygousPAM16mutation in a patient with a milder phenotype and longer survival. Am. J. Med. Genet. Part A.

[67] Davey K.M., Parboosingh J.S., McLeod D.R., Chan A., Casey R.A., Ferreira P.A., Snyder F.F., Bridge P., Bernier F.P. (2005). Mutation of DNAJC19, a human homologue of yeast inner mitochondrial membrane co-chaperones, causes DCMA syndrome, a novel autosomal recessive Barth syndrome-like condition. J. Med. Genet..

[68] Ojala T., Polinati P., Manninen T., Hiippala A., Rajantie J., Karikoski R., Suomalainen-Wartiovaara A., Tyni T. (2012). New mutation of mitochondrial DNAJC19 causing dilated and noncompaction cardiomyopathy, anemia, ataxia, and male genital anomalies. Pediatr. Res..

[69] Al Teneiji A., Siriwardena K., George K., Mital S., Mercimek-Mahmutoglu S. (2016). Progressive cerebellar atrophy and a novel homozygous pathogenic DNAJC19 variant as a cause of dilated cardiomyopathy ataxia syndrome. Pediatr. Neurol..

[70] Ucar S.K., Mayr J., Feichtinger R.G., Canda E., Çoker M., Wortmann S.B. (2016). Previously Unreported Biallelic Mutation in DNAJC19: Are Sensorineural Hearing Loss and Basal Ganglia Lesions Additional Features of Dilated Cardiomyopathy and Ataxia (DCMA) Syndrome?. JIMD Rep..

[71] Magen D., Georgopoulos C., Bross P., Ang D., Segev Y., Goldsher D., Nemirovski A., Shahar E., Ravid S., Luder A. (2008). Mitochondrial Hsp60 Chaperonopathy Causes an Autosomal-Recessive Neurodegenerative Disorder Linked to Brain Hypomyelination and Leukodystrophy. Am. J. Hum. Genet..

[72] Kusk M.S., Damgaard B., Risom L., Hansen B., Ostergaard E. (2016). Hypomyelinating Leukodystrophy due to HSPD1 Mutations: A New Patient. Neuropediatrics.

[73] Hansen J.J., Dürr A., Cournu-Rebeix I., Georgopoulos C., Ang D., Nielsen M.N., Davoine C.-S., Brice A., Fontaine B., Gregersen N. (2002). Hereditary Spastic Paraplegia SPG13 Is Associated with a Mutation in the Gene Encoding the Mitochondrial Chaperonin Hsp60. Am. J. Hum. Genet..

[74] Enomoto H., Mittal N., Inomata T., Arimura T., Izumi T., Kimura A., Fukuda K., Makino S. (2021). Dilated cardiomyopathy-linked heat shock protein family D member 1 mutations cause up-regulation of reactive oxygen species and autophagy through mitochondrial dysfunction. Cardiovasc. Res..

[75] Bie A.S., Fernandez-Guerra P., Birkler R.I.D., Nisemblat S., Pelnena D., Lu X., Deignan J.L., Lee H., Dorrani N., Corydon T.J. (2016). Effects of a Mutation in the HSPE1 Gene Encoding the Mitochondrial Co-chaperonin HSP10 and Its Potential Association with a Neurological and Developmental Disorder. Front. Mol. Biosci..

[76] Jobling R.K., Assoum M., Gakh O., Blaser S., Raiman J.A., Mignot C., Roze E., Durr A., Brice A., Lévy N. (2015). PMPCAmutations cause abnormal mitochondrial protein processing in patients with non-progressive cerebellar ataxia. Brain.

[77] Choquet K., Zurita-Rendón O., La Piana R., Yang S., Dicaire M.-J., Boycott K.M., Majewski J., Shoubridge E.A., Brais B., Tétreault M. (2015). Autosomal recessive cerebellar ataxia caused by a homozygous mutation in PMPCA. Brain.

[78] Joshi M., Anselm I., Shi J., Bale T.A., Towne M., Schmitz-Abe K., Crowley L., Giani F.C., Kazerounian S., Markianos K. (2016). Mutations in the substrate binding glycine-rich loop of the mitochondrial processing peptidase-α protein (PMPCA) cause a severe mitochondrial disease. Mol. Case Stud..

[79] Vögtle F.-N., Brändl B., Larson A., Pendziwiat M., Friederich M.W., White S.M., Basinger A., Kücükköse C., Muhle H., Jähn J.A. (2018). Mutations in PMPCB Encoding the Catalytic Subunit of the Mitochondrial Presequence Protease Cause Neurodegeneration in Early Childhood. Am. J. Hum. Genet..

[80] Eldomery M.K., Akdemir Z.C., Vögtle F.-N., Charng W.-L., Mulica P., Rosenfeld J.A., Gambin T., Gu S., Burrage L.C., Al Shamsi A. (2016). MIPEP recessive variants cause a syndrome of left ventricular non-compaction, hypotonia, and infantile death. Genome Med..

[81] Otto E.A., Ramaswami G., Janssen S., Chaki M., Allen S.J., Zhou W., Airik R., Hurd T.W., Ghosh A.K., Wolf M.T. (2010). Mutation analysis of 18 nephronophthisis associated ciliopathy disease genes using a DNA pooling and next generation sequencing strategy. J. Med. Genet..

[82] O’Toole J.F., Liu Y., Davis E., Westlake C.J., Attanasio M., Otto E.A., Seelow D., Nurnberg G., Becker C., Nuutinen M. (2010). Individuals with mutations in XPNPEP3, which encodes a mitochondrial protein, develop a nephronophthisis-like nephropathy. J. Clin. Investig..

[83] Alizadeh R., Jamshidi S., Keramatipour M., Moeinian P., Hosseini R., Otukesh H., Talebi S., Hospital T.A.A.C. (2020). Whole Exome Sequencing Reveals a XPNPEP3 Novel Mutation Causing Nephronophthisis in a Pediatric Patient. Iran. Biomed. J..

[84] Jin H., May M., Tranebjærg L., Kendall E., Fontán G., Jackson J., Subramony S., Arena F., Lubs H., Smith S. (1996). A novel X–linked gene, DDP, shows mutations in families with deafness (DFN–1), dystonia, mental deficiency and blindness. Nat. Genet..

[85] Swerdlow R.H., Wooten G.F. (2001). A novel deafness/dystonia peptide gene mutation that causes dystonia in female carriers of Mohr-Tranebjaerg syndrome. Ann. Neurol..

[86] Aguirre L.A., Del Castillo I., Macaya A., Medá C., Villamar M., Moreno-Pelayo M.A., Moreno F. (2006). A novel mutation in the gene encoding TIMM8a, a component of the mitochondrial protein translocase complexes, in a Spanish familial case of deafness-dystonia (Mohr–Tranebjaerg) syndrome. Am. J. Med. Genet. Part. A.

[87] Tranebjaerg L., Van Ghelue M., Nilssen O., Hodes M.E., Dlouhy S.R., Farlow M.R., Hamel B., Arts W.F.M., Jankovic J., Beach J. (1997). Jensen syndrome is allelic to Mohr–Tranebjaerg syndrome and both are caused by stop mutations in the DDP gene. Am. J. Hum. Genet..

[88] Ujike H., Tanabe Y., Takehisa Y., Hayabara T., Kuroda S. (2001). A Family With X-linked Dystonia-Deafness Syndrome with a Novel Mutation of the DDP Gene. Arch. Neurol..

[89] Blesa J.R., Solano A., Briones P., Prieto-Ruiz J.A., Hernández-Yago J., Coria F. (2007). Molecular Genetics of a Patient with Mohr–Tranebjaerg Syndrome due to a New Mutation in the DDP1 Gene. NeuroMolecular Med..

[90] Song P., Guan Y., Chen X., Wu C., Qiao A., Jiang H., Li Q., Huang Y., Huang W., Xu M. (2020). Frameshift mutation of Timm8a1 gene in mouse leads to an abnormal mitochondrial structure in the brain, correlating with hearing and memory impairment. J. Med. Genet..

[91] Tranebjærg L., Hamel B.C., Gabreels F.J., O Renier W., Van Ghelue M. (2000). A de novo missense mutation in a critical domain of the X-linked DDP gene causes the typical deafness–dystonia–optic atrophy syndrome. Eur. J. Hum. Genet..

[92] Binder J., Hofmann S., Kreisel S., Wöhrle J.C., Bäzner H., Krauss J.K., Hennerici M.G., Bauer M.F. (2003). Clinical and molecular findings in a patient with a novel mutation in the deafness-dystonia peptide (DDP1) gene. Brain.

[93] Ezquerra M., Campdelacreu J., Muñoz E., Tolosa E., Martí M.J. (2005). A Novel Intronic Mutation in the DDP1 Gene in a Family With X-linked Dystonia-Deafness Syndrome. Arch. Neurol..

[94] Kim H.T., Edwards M.J., Tyson J., Quinn N.P., Bitner-Glindzicz M., Bhatia K.P. (2007). Blepharospasm and limb dystonia caused by Mohr-Tranebjaerg syndrome with a novel splice-site mutation in the deafness/dystonia peptide gene. Mov. Disord..

[95] Aguirre L.A., Pérez-Bas M., Villamar M., López-Ariztegui M.A., Moreno-Pelayo M.A., Moreno F., Del Castillo I. (2008). A Spanish sporadic case of deafness–dystonia (Mohr-Tranebjaerg) syndrome with a novel mutation in the gene encoding TIMM8a, a component of the mitochondrial protein translocase complexes. Neuromuscul. Disord..

[96] Pizzuti A., Fabbrini G., Salehi L., Vacca L., Inghilleri M., Dallapiccola B., Berardelli A. (2004). Focal dystonia caused by Mohr-Tranebjaerg syndrome with complete deletion of the DDP1 gene. Neurology.

[97] Richter D., Conley M.E., Rohrer J., Myers L.A., Zahradka K., Ic J.K., Sertic J., Rukavina A.S. (2001). A contiguous deletion syndrome of X-linked agammaglobulinemia and sensorineural deafness. Pediatr. Allergy Immunol..

[98] Šedivá A., Smith C.I.E., Asplund A.C., Hadač J., Janda A., Zeman J., Hansíková H., Dvořáková L., Mrázová L., Velbri S. (2007). Contiguous X-chromosome Deletion Syndrome Encompassing the BTK, TIMM8A, TAF7L, and DRP2 Genes. J. Clin. Immunol..

[99] Arai T., Zhao M., Kanegane H., Van Zelm M.C., Futatani T., Yamada M., Ariga T., Ochs H.D., Miyawaki T., Oh-Ishi T. (2011). Genetic analysis of contiguous X-chromosome deletion syndrome encompassing the BTK and TIMM8A genes. J. Hum. Genet..

[100] Calvo S.E., Compton A., Hershman S., Lim S.C., Lieber D.S., Tucker E., Laskowski A., Garone C., Liu S., Jaffe D.B. (2012). Molecular Diagnosis of Infantile Mitochondrial Disease with Targeted Next-Generation Sequencing. Sci. Transl. Med..

[101] Mayr J.A., Haack T.B., Graf E., Zimmermann F.A., Wieland T., Haberberger B., Superti-Furga A., Kirschner J., Steinmann B., Baumgartner M.R. (2012). Lack of the Mitochondrial Protein Acylglycerol Kinase Causes Sengers Syndrome. Am. J. Hum. Genet..

[102] Siriwardena K., MacKay N., Levandovskiy V., Blaser S., Raiman J., Kantor P., Ackerley C., Robinson B.H., Schulze A., Cameron J.M. (2013). Mitochondrial citrate synthase crystals: Novel finding in Sengers syndrome caused by acylglycerol kinase (AGK) mutations. Mol. Genet. Metab..

[103] Haghighi A., Haack T.B., Atiq M., Mottaghi H., Haghighi-Kakhki H., Bashir R.A., Ahting U., Feichtinger R.G., Mayr J.A., Rötig A. (2014). Sengers syndrome: Six novel AGK mutations in seven new families and review of the phenotypic and mutational spectrum of 29 patients. Orphanet J. Rare Dis..

[104] Allali S., Dorboz I., Samaan S., Slama A., Rambaud C., Boespflug-Tanguy O., Sarret C. (2017). Mutation in the AGK gene in two siblings with unusual Sengers syndrome. Metab. Brain Dis..

[105] Guleray N., Kosukcu C., Taskiran Z.E., Simsek Kiper P.O., Utine G.E., Gucer S., Tokatli A., Boduroglu K., Alikasifoglu M. (2019). Atypical Presentation of Sengers Syndrome: A Novel Mutation Revealed with Postmortem Genetic Testing. Fetal Pediatr. Pathol..

[106] Aldahmesh M.A., Khan A.O., Mohamed J.Y., Alghamdi M.H., Alkuraya F.S. (2012). Identification of a truncation mutation of acylglycerol kinase (AGK) gene in a novel autosomal recessive cataract locus. Hum. Mutat..

[107] Pacheu-Grau D., Callegari S., Emperador S., Thompson K., Aich A., E Topol S., Spencer E.G., McFarland R., Ruiz-Pesini E., Torkamani A. (2018). Mutations of the mitochondrial carrier translocase channel subunit TIM22 cause early-onset mitochondrial myopathy. Hum. Mol. Genet..

[108] Guarani V., Jardel C., Chrétien D., Lombès A., Bénit P., Labasse C., Lacène E., Bourillon A., Imbard A., Benoist J.-F. (2016). QIL1 mutation causes MICOS disassembly and early onset fatal mitochondrial encephalopathy with liver disease. eLife.

[109] Zeharia A., Friedman J., Tobar A., Saada A., Konen O., Fellig Y., Shaag A., Nunnari J., Elpeleg O. (2016). Mitochondrial hepato-encephalopathy due to deficiency of QIL1/MIC13 (C19orf70), a MICOS complex subunit. Eur. J. Hum. Genet..

[110] Kishita Y., Shimura M., Kohda M., Akita M., Imai-Okazaki A., Yatsuka Y., Nakajima Y., Ito T., Ohtake A., Murayama K. (2020). A novel homozygous variant in MICOS13/QIL1 causes hepato-encephalopathy with mitochondrial DNA depletion syndrome. Mol. Genet. Genom. Med..

[111] Gödiker J., Grüneberg M., Duchesne I., Reunert J., Rust S., Westermann C., Wada Y., Classen G., Langhans C.D., Schlingmann K.P. (2018). QIL1-dependent assembly of MICOS complex–lethal mutation in C19ORF70 resulting in liver disease and severe neurological retardation. J. Hum. Genet..

[112] Benincá C., Zanette V., Brischigliaro M., Johnson M., Reyes A., Valle D.A.D., Robinson A.J., Degiorgi A., Yeates A., Telles B.A. (2021). Mutation in the MICOS subunit gene APOO (MIC26) associated with an X-linked recessive mitochondrial myopathy, lactic acidosis, cognitive impairment and autistic features. J. Med. Genet..

[113] Elouej S., Harhouri K., Le Mao M., Baujat G., Nampoothiri S., Kayserili H., Al Menabawy N., Selim L., Paneque A.L., Kubisch C. (2020). Loss of MTX2 causes mandibuloacral dysplasia and links mitochondrial dysfunction to altered nuclear morphology. Nat. Commun..

[114] Chacinska A., Pfannschmidt S., Wiedemann N., Kozjak V., Sanjuán Szklarz L.K., Schulze-Specking A., Truscott K.N., Guiard B., Meisinger C., Pfanner N. (2004). Essential role of Mia40 in import and assembly of mitochondrial intermembrane space proteins. EMBO J..

[115] Müller J.M., Milenkovic D., Guiard B., Pfanner N., Chacinska A. (2008). Precursor Oxidation by Mia40 and Erv1 Promotes Vectorial Transport of Proteins into the Mitochondrial Intermembrane Space. Mol. Biol. Cell.

[116] Sztolsztener M.E., Brewinska A., Guiard B., Chacinska A. (2013). Disulfide Bond Formation: Sulfhydryl Oxidase ALR Controls Mitochondrial Biogenesis of Human MIA40. Traffic.

[117] Peleh V., Cordat E., Herrmann J.M. (2016). Mia40 is a trans-site receptor that drives protein import into the mitochondrial intermembrane space by hydrophobic substrate binding. eLife.

[118] Mesecke N., Terziyska N., Kozany C., Baumann F., Neupert W., Hell K., Herrmann J.M. (2005). A Disulfide Relay System in the Intermembrane Space of Mitochondria that Mediates Protein Import. Cell.

[119] Banci L., Bertini I., Cefaro C., Baffoni S.C., Gallo A., Martinelli M., Sideris D.P., Katrakili N., Tokatlidis K. (2009). MIA40 is an oxidoreductase that catalyzes oxidative protein folding in mitochondria. Nat. Struct. Mol. Biol..

[120] Fischer M., Horn S., Belkacemi A., Kojer K., Petrungaro C., Habich M., Ali M., Küttner V., Bien M., Kauff F. (2013). Protein import and oxidative folding in the mitochondrial intermembrane space of intact mammalian cells. Mol. Biol. Cell.

[121] Meyer K., Buettner S., Ghezzi D., Zeviani M., Bano D., Nicotera P. (2015). Loss of apoptosis-inducing factor critically affects MIA40 function. Cell Death Dis..

[122] Hangen E., Féraud O., Lachkar S., Mou H., Doti N., Fimia G.M., Lam N.-V., Zhu C., Godin I., Muller K. (2015). Interaction between AIF and CHCHD4 Regulates Respiratory Chain Biogenesis. Mol. Cell.

[123] Susin S.A., Lorenzo H.K., Zamzami N., Marzo I., Snow B.E., Brothers G.M., Mangion J., Jacotot E., Costantini P., Loeffler M. (1999). Molecular characterization of mitochondrial apoptosis-inducing factor. Nature.

[124] Vahsen N., Candé C., Brière J.-J., Bénit P., Joza N., Larochette N., Mastroberardino P.G., O Pequignot M., Casares N., Lazar V. (2004). AIF deficiency compromises oxidative phosphorylation. EMBO J..

[125] Joza N., Oudit G.Y., Brown D., Bénit P., Kassiri Z., Vahsen N., Benoit L., Patel M.M., Nowikovsky K., Vassault A. (2005). Muscle-Specific Loss of Apoptosis-Inducing Factor Leads to Mitochondrial Dysfunction, Skeletal Muscle Atrophy, and Dilated Cardiomyopathy. Mol. Cell. Biol..

[126] Brown D., Yu B., Joza N., Bénit P., Meneses J., Firpo M., Rustin P., Penninger J.M., Martin G.R. (2006). Loss of Aif function causes cell death in the mouse embryo, but the temporal progression of patterning is normal. Proc. Natl. Acad. Sci. USA.

[127] Modjtahedi N., Tokatlidis K., Dessen P., Kroemer G. (2016). Mitochondrial Proteins Containing Coiled-Coil-Helix-Coiled-Coil-Helix (CHCH) Domains in Health and Disease. Trends Biochem. Sci..

[128] Cavallaro G. (2010). Genome-wide analysis of eukaryotic twin CX9C proteins. Mol. BioSyst..

[129] Petrungaro C., Zimmermann K.M., Küttner V., Fischer M., Dengjel J., Bogeski I., Riemer J. (2015). The Ca2+-Dependent Release of the Mia40-Induced MICU1-MICU2 Dimer from MCU Regulates Mitochondrial Ca^2+^ Uptake. Cell Metab..

[130] Friederich M.W., Erdogan A.J., Coughlin C.R., Elos M.T., Jiang H., O’Rourke C.P., Lovell M.A., Wartchow E., Gowan K., Chatfield K.C. (2016). Mutations in the accessory subunitNDUFB10result in isolated complex I deficiency and illustrate the critical role of intermembrane space import for complex I holoenzyme assembly. Hum. Mol. Genet..

[131] Bano D., Prehn J.H. (2018). Apoptosis-Inducing Factor (AIF) in Physiology and Disease: The Tale of a Repented Natural Born Killer. EBioMedicine.

[132] Sevrioukova I.F. (2016). Structure/Function Relations in AIFM1 Variants Associated with Neurodegenerative Disorders. J. Mol. Biol..

[133] Wischhof L., Gioran A., Sonntag-Bensch D., Piazzesi A., Stork M., Nicotera P., Bano D. (2018). A disease-associated Aifm1 variant induces severe myopathy in knockin mice. Mol. Metab..

[134] Troulinaki K., Büttner S., Cots A.M., Maida S., Meyer K., Bertan F., Gioran A., Piazzesi A., Fornarelli A., Nicotera P. (2018). WAH-1/AIF regulates mitochondrial oxidative phosphorylation in the nematode Caenorhabditis elegans. Cell Death Discov..

[135] Vögtle F.-N., Wortelkamp S., Zahedi R., Becker D., Leidhold C., Gevaert K., Kellermann J., Voos W., Sickmann A., Pfanner N. (2009). Global Analysis of the Mitochondrial N-Proteome Identifies a Processing Peptidase Critical for Protein Stability. Cell.

[136] Vögtle F.-N., Prinz C., Kellermann J., Lottspeich F., Pfanner N., Meisinger C. (2011). Mitochondrial protein turnover: Role of the precursor intermediate peptidase Oct1 in protein stabilization. Mol. Biol. Cell.

[137] Veling M.T., Reidenbach A.G., Freiberger E.C., Kwiecien N.W., Hutchins P.D., Drahnak M.J., Jochem A., Ulbrich A., Rush M.J., Russell J.D. (2017). Multi-omic Mitoprotease Profiling Defines a Role for Oct1p in Coenzyme Q Production. Mol. Cell.

[138] Ostermann J., Horwich A.L., Neupert W., Hartl F.-U. (1989). Protein folding in mitochondria requires complex formation with hsp60 and ATP hydrolysis. Nat. Cell Biol..

[139] Höhfeld J., Hartl F.U. (1994). Role of the chaperonin cofactor Hsp10 in protein folding and sorting in yeast mitochondria. J. Cell Biol..

[140] Bauer M.F., Gempel K., Reichert A., Rappold G.A., Lichtner P., Gerbitz K.-D., Neupert W., Brunner M., Hofmann S. (1999). Genetic and structural characterization of the human mitochondrial inner membrane translocase. J. Mol. Biol..

[141] Guo Y., Cheong N., Zhang Z., De Rose R., Deng Y., Farber S.A., Fernandes-Alnemri T., Alnemri E.S. (2004). Tim50, a Component of the Mitochondrial Translocator, Regulates Mitochondrial Integrity and Cell Death. J. Biol. Chem..

[142] Sinha D., Srivastava S., Krishna L., D’Silva P. (2014). Unraveling the Intricate Organization of Mammalian Mitochondrial Presequence Translocases: Existence of Multiple Translocases for Maintenance of Mitochondrial Function. Mol. Cell. Biol..

[143] Truscott K., Kovermann P., Geissler A., Merlin A., Meijer M., Driessen A.J., Rassow J., Pfanner N., Wagner R. (2001). A presequence- and voltage-sensitive channel of the mitochondrial preprotein translocase formed by Tim23. Nat. Genet..

[144] Zhou S., Ruan M., Li Y., Yang J., Bai S., Richter C., Schwalbe H., Xie C., Shen B., Wang J. (2020). Solution structure of the voltage-gated Tim23 channel in complex with a mitochondrial presequence peptide. Cell Res..

[145] Bauer M.F., Sirrenberg C., Neupert W., Brunner M. (1996). Role of Tim23 as Voltage Sensor and Presequence Receptor in Protein Import into Mitochondria. Cell.

[146] Meinecke M. (2006). Tim50 Maintains the Permeability Barrier of the Mitochondrial Inner Membrane. Science.

[147] Martin J., Mahlke K., Pfanner N. (1991). Role of an energized inner membrane in mitochondrial protein import. Delta psi drives the movement of presequences. J. Biol. Chem..

[148] Denkert N., Schendzielorz A.B., Barbot M., Versemann L., Richter F., Rehling P., Meinecke M. (2017). Cation selectivity of the presequence translocase channel Tim23 is crucial for efficient protein import. eLife.

[149] Bhattacharyya T., Karnezis A.N., Murphy S.P., Hoang T., Freeman B.C., Phillips B., Morimoto R.I. (1995). Cloning and Subcellular Localization of Human Mitochondrial hsp70. J. Biol. Chem..

[150] Borges J., Fischer H., Craievich A., Hansen L.D., Ramos C. (2003). Free Human Mitochondrial GrpE Is a Symmetric Dimer in Solution. J. Biol. Chem..

[151] Chacinska A., Lind M., Frazier A., Dudek J., Meisinger C., Geissler A., Sickmann A., Meyer H.E., Truscott K.N., Guiard B. (2005). Mitochondrial Presequence Translocase: Switching between TOM Tethering and Motor Recruitment Involves Tim21 and Tim17. Cell.

[152] Sinha D., Joshi N., Chittoor B., Samji P., D’Silva P. (2010). Role of Magmas in protein transport and human mitochondria biogenesis. Hum. Mol. Genet..

[153] Mick D., Dennerlein S., Wiese H., Reinhold R., Pacheu-Grau D., Lorenzi I., Sasarman F., Weraarpachai W., Shoubridge E.A., Warscheid B. (2012). MITRAC Links Mitochondrial Protein Translocation to Respiratory-Chain Assembly and Translational Regulation. Cell.

[154] Sinha D., Srivastava S., D’Silva P. (2016). Functional Diversity of Human Mitochondrial J-proteins Is Independent of Their Association with the Inner Membrane Presequence Translocase. J. Biol. Chem..

[155] Richter F., Dennerlein S., Nikolov M., Jans D.C., Naumenko N., Aich A., MacVicar T., Linden A., Jakobs S., Urlaub H. (2019). ROMO1 is a constituent of the human presequence translocase required for YME1L protease import. J. Cell Biol..

[156] Dennerlein S., Rehling P. (2015). Human mitochondrial COX1 assembly into cytochrome c oxidase at a glance. J. Cell Sci..

[157] Richter-Dennerlein R., Oeljeklaus S., Lorenzi I., Ronsör C., Bareth B., Schendzielorz A.B., Wang C., Warscheid B., Rehling P., Dennerlein S. (2016). Mitochondrial Protein Synthesis Adapts to Influx of Nuclear-Encoded Protein. Cell.

[158] Rainbolt T., Atanassova N., Genereux J.C., Wiseman R.L. (2013). Stress-Regulated Translational Attenuation Adapts Mitochondrial Protein Import through Tim17A Degradation. Cell Metab..

[159] MacVicar T., Ohba Y., Nolte H., Mayer F.C., Tatsuta T., Sprenger H.-G., Lindner B., Zhao Y., Li J., Bruns C. (2019). Lipid signalling drives proteolytic rewiring of mitochondria by YME1L. Nature.

[160] Richter-Dennerlein R., Korwitz A., Haag M., Tatsuta T., Dargazanli S., Baker M., Decker T., Lamkemeyer T., Rugarli E., Langer T. (2014). DNAJC19, a Mitochondrial Cochaperone Associated with Cardiomyopathy, Forms a Complex with Prohibitins to Regulate Cardiolipin Remodeling. Cell Metab..

[161] Rehling P., Brandner K., Pfanner N. (2004). Mitochondrial import and the twin-pore translocase. Nat. Rev. Mol. Cell Biol..

[162] Pfanner N., Neupert W. (1987). Distinct steps in the import of ADP/ATP carrier into mitochondria. J. Biol. Chem..

[163] Zara V., Palmisano I., Rassow J., Palmieri F. (2001). Biogenesis of the dicarboxylate carrier (DIC): Translocation across the mitochondrial outer membrane and subsequent release from the TOM channel are membrane potential-independent. J. Mol. Biol..

[164] Hasson S., Damoiseaux R., Glavin J.D., Dabir D.V., Walker S.S., Koehler C.M. (2010). Substrate specificity of the TIM22 mitochondrial import pathway revealed with small molecule inhibitor of protein translocation. Proc. Natl. Acad. Sci. USA.

[165] Callegari S., Richter F., Chojnacka K., Jans D.C., Lorenzi I., Pacheu-Grau D., Jakobs S., Lenz C., Urlaub H., Dudek J. (2016). TIM29 is a subunit of the human carrier translocase required for protein transport. FEBS Lett..

[166] Kang Y., Baker M.J., Liem M., Louber J., McKenzie M., Atukorala I., Ang C.-S., Keerthikumar S., Mathivanan S., Stojanovski D. (2016). Tim29 is a novel subunit of the human TIM22 translocase and is involved in complex assembly and stability. eLife.

[167] Kang Y., Stroud D., Baker M.J., DE Souza D., Frazier A.E., Liem M., Tull D., Mathivanan S., McConville M.J., Thorburn D. (2017). Sengers Syndrome-Associated Mitochondrial Acylglycerol Kinase Is a Subunit of the Human TIM22 Protein Import Complex. Mol. Cell.

[168] Vukotic M., Nolte H., König T., Saita S., Ananjew M., Krüger M., Tatsuta T., Langer T. (2017). Acylglycerol Kinase Mutated in Sengers Syndrome Is a Subunit of the TIM22 Protein Translocase in Mitochondria. Mol. Cell.

[169] Gomkale R., Cruz-Zaragoza L.D., Suppanz I., Guiard B., Montoya J., Callegari S., Pacheu-Grau D., Warscheid B., Rehling P. (2020). Defining the Substrate Spectrum of the TIM22 Complex Identifies Pyruvate Carrier Subunits as Unconventional Cargos. Curr. Biol..

[170] Rampelt H., Sucec I., Bersch B., Horten P., Perschil I., Martinou J.-C., Van Der Laan M., Wiedemann N., Schanda P., Pfanner N. (2020). The mitochondrial carrier pathway transports non-canonical substrates with an odd number of transmembrane segments. BMC Biol..

[171] Jackson T.D., Hock D.H., Fujihara K.M., Palmer C.S., Frazier A.E., Low Y.C., Kang Y., Ang C.-S., Clemons N.J., Thorburn D.R. (2021). The TIM22 complex mediates the import of sideroflexins and is required for efficient mitochondrial one-carbon metabolism. Mol. Biol. Cell.

[172] Beverly K.N., Sawaya M.R., Schmid E., Koehler C.M. (2008). The Tim8–Tim13 Complex Has Multiple Substrate Binding Sites and Binds Cooperatively to Tim23. J. Mol. Biol..

[173] Gebert N., Chacinska A., Wagner K., Guiard B., Koehler C.M., Rehling P., Pfanner N., Wiedemann N. (2008). Assembly of the three small Tim proteins precedes docking to the mitochondrial carrier translocase. EMBO Rep..

[174] Weinhäupl K., Lindau C., Hessel A., Wang Y., Schütze C., Jores T., Melchionda L., Schönfisch B., Kalbacher H., Bersch B. (2018). Structural Basis of Membrane Protein Chaperoning through the Mitochondrial Intermembrane Space. Cell.

[175] Valpadashi A., Callegari S., Linden A., Neumann P., Ficner R., Urlaub H., Deckers M., Rehling P. (2021). Defining the architecture of the human TIM22 complex by chemical crosslinking. FEBS Lett..

[176] Zhang Y., Ou X., Wang X., Sun D., Zhou X., Wu X., Li Q., Li L. (2021). Structure of the mitochondrial TIM22 complex from yeast. Cell Res..

[177] Qi L., Wang Q., Guan Z., Wu Y., Shen C., Hong S., Cao J., Zhang X., Yan C., Yin P. (2021). Cryo-EM structure of the human mitochondrial translocase TIM22 complex. Cell Res..

[178] Kang Y., Anderson A.J., Jackson T.D., Palmer C.S., De Souza D.P., Fujihara K.M., Stait T., Frazier A., Clemons N.J., Tull D. (2019). Function of hTim8a in complex IV assembly in neuronal cells provides insight into pathomechanism underlying Mohr-Tranebjærg syndrome. eLife.

[179] Rehling P. (2003). Protein Insertion into the Mitochondrial Inner Membrane by a Twin-Pore Translocase. Science.

[180] Kovermann P., Truscott K.N., Guiard B., Rehling P., Sepuri N.B., Müller H., E Jensen R., Wagner R., Pfanner N. (2002). Tim22, the Essential Core of the Mitochondrial Protein Insertion Complex, Forms a Voltage-Activated and Signal-Gated Channel. Mol. Cell.

[181] Callegari S., Müller T., Schulz C., Lenz C., Jans D.C., Wissel M., Opazo F., Rizzoli S.O., Jakobs S., Urlaub H. (2019). A MICOS–TIM22 Association Promotes Carrier Import into Human Mitochondria. J. Mol. Biol..

[182] Ellenrieder L., Dieterle M.P., Doan K.N., Mårtensson C.U., Floerchinger A., Campo M.L., Pfanner N., Becker T. (2019). Dual Role of Mitochondrial Porin in Metabolite Transport across the Outer Membrane and Protein Transfer to the Inner Membrane. Mol. Cell.

[183] Sakaue H., Shiota T., Ishizaka N., Kawano S., Tamura Y., Tan K.S., Imai K., Motono C., Hirokawa T., Taki K. (2019). Porin Associates with Tom22 to Regulate the Mitochondrial Protein Gate Assembly. Mol. Cell.

[184] Mohr J., Mageröy K. (1960). Sex-Linked Deafness of a Possibly New Type. Hum. Hered..

[185] Tranebjaerg L., Schwartz C., Eriksen H., Andreasson S., Ponjavic V., Dahl A., Stevenson R.E., May M., Arena F., Barker D. (1995). A new X linked recessive deafness syndrome with blindness, dystonia, fractures, and mental deficiency is linked to Xq22. J. Med. Genet..

[186] Paschen S.A., Rothbauer U., Káldi K., Bauer M.F., Neupert W., Brunner M. (2000). The role of the TIM8-13 complex in the import of Tim23 into mitochondria. EMBO J..

[187] Rothbauer U., Hofmann S., Mühlenbein N., Paschen S.A., Gerbitz K.-D., Neupert W., Brunner M., Bauer M.F. (2001). Role of the Deafness Dystonia Peptide 1 (DDP1) in Import of Human Tim23 into the Inner Membrane of Mitochondria. J. Biol. Chem..

[188] Roesch K., Curran S.P., Tranebjaerg L., Koehler C.M. (2002). Human deafness dystonia syndrome is caused by a defect in assembly of the DDP1/TIMM8a-TIMM13 complex. Hum. Mol. Genet..

[189] Sengers R., Trijbels J., Willems J., Daniels O., Stadhouders A. (1975). Congenital cataract and mitochondrial myopathy of skeletal and heart muscle associated with lactic acidosis after exercise. J. Pediatr..

[190] Jordens E.Z., Palmieri L., Huizing M., Heuvel L.P.V.D., Sengers R.C.A., Dörner A., Ruitenbeek W., Trijbels F.J., Valsson J., Sigfusson G. (2002). Adenine nucleotide translocator 1 deficiency associated with Sengers syndrome. Ann. Neurol..

[191] Khan A.O., Aldahmesh M.A., Alkuraya F.S. (2015). Phenotypes of Recessive Pediatric Cataract in a Cohort of Children with Identified Homozygous Gene Mutations (An American Ophthalmological Society Thesis). Trans. Am. Ophthalmol. Soc..

[192] Becker T., Wagner R. (2018). Mitochondrial Outer Membrane Channels: Emerging Diversity in Transport Processes. BioEssays.

[193] Paschen S.A., Waizenegger T., Stan T., Preuss M., Cyrklaff M., Hell K., Rapaport D., Neupert W. (2003). Evolutionary conservation of biogenesis of β-barrel membrane proteins. Nat. Cell Biol..

[194] Wiedemann N., Kozjak V., Chacinska A., Schönfisch B., Rospert S., Ryan M.T., Pfanner N., Meisinger C. (2003). Machinery for protein sorting and assembly in the mitochondrial outer membrane. Nat. Cell Biol..

[195] Kozjak-Pavlovic V., Ross K., Benlasfer N., Kimmig S., Karlas A., Rudel T. (2007). Conserved roles of Sam50 and metaxins in VDAC biogenesis. EMBO Rep..

[196] Bohnert M., Wenz L.-S., Zerbes R.M., Horvath S.E., Stroud D., Von Der Malsburg K., Mueller J.M., Oeljeklaus S., Perschil I., Warscheid B. (2012). Role of mitochondrial inner membrane organizing system in protein biogenesis of the mitochondrial outer membrane. Mol. Biol. Cell.

[197] Dimmer K.S., Papić D., Schumann B., Sperl D., Krumpe K., Walther D.M., Rapaport D. (2012). A crucial role of Mim2 in the biogenesis of mitochondrial outer membrane proteins. J. Cell Sci..

[198] Papić D., Krumpe K., Dukanovic J., Dimmer K.S., Rapaport D. (2011). Multispan mitochondrial outer membrane protein Ugo1 follows a unique Mim1-dependent import pathway. J. Cell Biol..

[199] Popov-Čeleketić J., Waizenegger T., Rapaport D. (2008). Mim1 Functions in an Oligomeric Form to Facilitate the Integration of Tom20 into the Mitochondrial Outer Membrane. J. Mol. Biol..

[200] Vögtle F.-N., Keller M., Taskin A.A., Horvath S.E., Guan X.L., Prinz C., Opalińska M., Zorzin C., Van Der Laan M., Wenk M.R. (2015). The fusogenic lipid phosphatidic acid promotes the biogenesis of mitochondrial outer membrane protein Ugo1. J. Cell Biol..

[201] Keskin A., Akdoğan E., Dunn C.D. (2017). Evidence for Amino Acid Snorkeling from a High-Resolution, In Vivo Analysis of Fis1 Tail-Anchor Insertion at the Mitochondrial Outer Membrane. Genetics.

[202] Endo T., Sakaue H. (2019). Multifaceted roles of porin in mitochondrial protein and lipid transport. Biochem. Soc. Trans..

[203] Grevel A., Becker T. (2019). Porins as helpers in mitochondrial protein translocation. Biol. Chem..

[204] Palmer C.S., Anderson A.J., Stojanovski D. (2021). Mitochondrial protein import dysfunction: Mitochondrial disease, neurodegenerative disease and cancer. FEBS Lett..

